# Stepwise assembly of multiple Lin28 proteins on the terminal loop of let-7 miRNA precursors

**DOI:** 10.1093/nar/gkt1391

**Published:** 2014-01-21

**Authors:** Alexandre Desjardins, Jonathan Bouvette, Pascale Legault

**Affiliations:** Département de Biochimie et Médecine Moléculaire, Université de Montréal, C.P. 6128, Succursale Centre-Ville, Montréal, QC H3C 3J7, Canada

## Abstract

Lin28 inhibits the biogenesis of let-7 miRNAs through direct interactions with let-7 precursors. Previous studies have described seemingly inconsistent Lin28 binding sites on pre-let-7 RNAs. Here, we reconcile these data by examining the binding mechanism of Lin28 to the terminal loop of pre-let-7g (TL-let-7g) using biochemical and biophysical methods. First, we investigate Lin28 binding to TL-let-7g variants and short RNA fragments and identify three independent binding sites for Lin28 on TL-let-7g. We then determine that Lin28 assembles in a stepwise manner on TL-let-7g to form a stable 1:3 complex. We show that the cold-shock domain (CSD) of Lin28 is responsible for remodelling the terminal loop of TL-let-7g, whereas the NCp7-like domain facilitates the initial binding of Lin28 to TL-let-7g. This stable binding of multiple Lin28 molecules to the terminal loop of pre-let-7g extends to other precursors of the let-7 family, but not to other pre-miRNAs tested. We propose a model for stepwise assembly of the 1:1, 1:2 and 1:3 pre-let-7g/Lin28 complexes. Stepwise multimerization of Lin28 on pre-let-7 is required for maximum inhibition of Dicer cleavage for a least one member of the let-7 family and may be important for orchestrating the activity of the several factors that regulate let-7 biogenesis.

## INTRODUCTION

MicroRNAs (miRNAs) are short single-stranded RNAs that control fundamental biological processes in plants and animals by acting as post-transcriptional regulators of mRNA expression [for detailed reviews see (1–4)]. The miRNAs from the let-7 family were among the first to be identified and have been extensively studied given their central roles in development, cell differentiation and tumour suppression (5–13). The let-7 miRNA loci are often present in multiple copies in a single genome, with the mature let-7 miRNAs being highly conserved across species (6,7,13,14). Furthermore, the terminal loops of their precursors contain highly conserved nucleotides that mediate interaction with factors that control miRNA biogenesis, such as Lin28 (15–19), hnRNP A1 (20,21) and the KH-type splicing regulatory protein (KSRP) (21–23), thereby adding a layer of complexity to miRNA-mediated gene regulation.

The Lin28 protein is a key post-transcriptional inhibitor of miRNA biogenesis that acts selectively on let-7 miRNAs (16,24), and there exists two isoforms in mammals [Lin28A (also termed Lin28) and Lin28B (25)]. Lin28 inhibits Drosha cleavage of primary let-7 transcripts (pri-let-7) in the nucleus (16,24,26,27) and interferes with Dicer cleavage of precursor let-7 (pre-let-7) in the cytoplasm (18,28,29). Moreover, Lin28 can counteract the stimulation of let-7 biogenesis brought about by KSRP, a factor that directly interacts with the terminal loops of several pri/pre-let-7 miRNAs (22,23). In the absence of Lin28, several Tutases (TUT2, TUT4 and TUT7) mono-uridylate a specific subset of pre-miRNAs, and this enhances the cleavage activity of Dicer (30). In contrast, Lin28 induces the oligo-uridylation of pre-let-7 by terminal uridylyl transferases (TUT4/Zcchc11 or TUT7/Zcchc6 in mouse and human), which inhibits cleavage by Dicer and promotes pre-let-7 decay (17,28,31–35). Thus, Lin28 negatively regulates let-7 biogenesis by inhibiting the Drosha- and Dicer-mediated cleavage of immature forms of let-7, by counteracting the action of factors that promote such processing and by enhancing mechanisms that specifically promote pre-let-7 decay.

Lin28 also regulates translation of several mRNAs [for a recent review see (36)] and functions as one of four factors that are sufficient to reprogram human somatic cells into induced pluripotent stem cells (37). Not surprisingly, recent studies have also associated Lin28 with development traits (38–40), development defects (41,42), tissue repair (43), increased cancer susceptibility (44–47) and advanced human malignancies (47–49).

**Figure 1.**
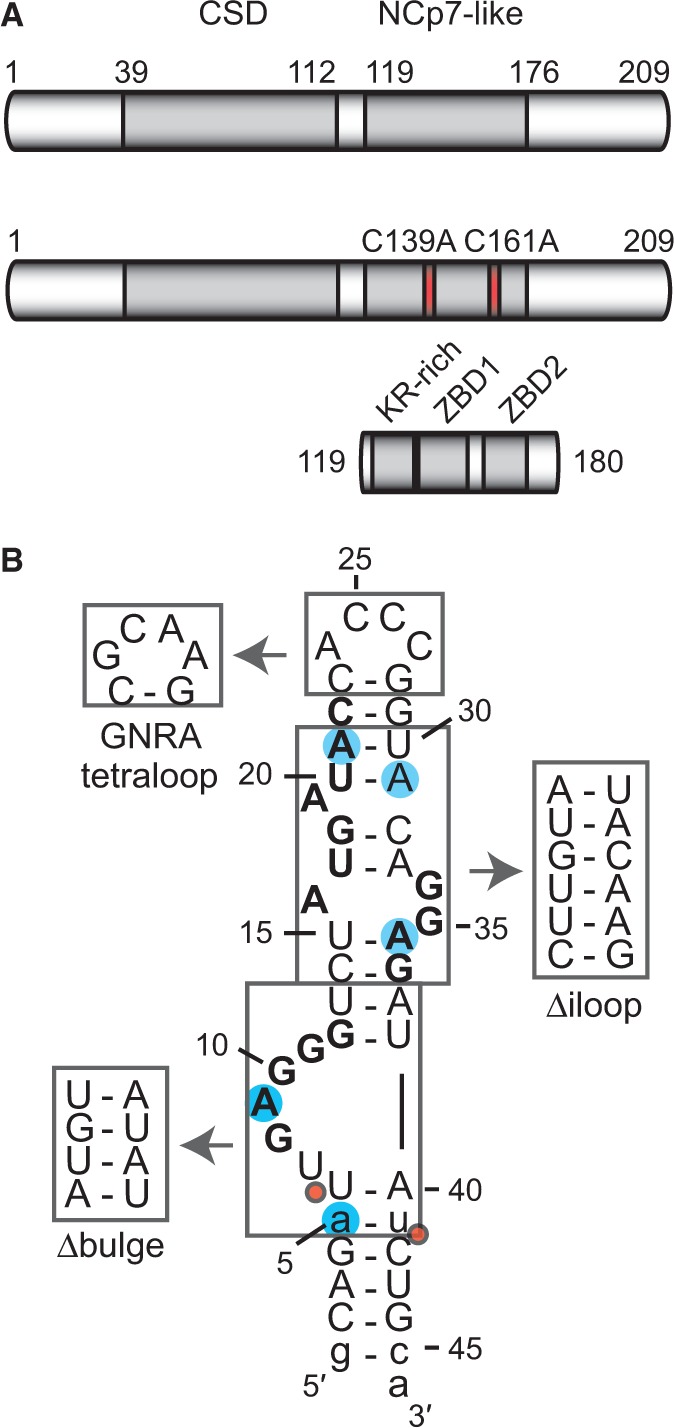
The Lin28 protein, TL-let-7g RNA and related sequences used in this study. (**A**) Schematic representation of the primary structures of Lin28 and related variants, Lin28 C139A/C161A and Lin28_119–180_. The grey boxes delineate sequences of known RNA-binding motifs: a CSD and an NCp7-like domain with a KR-rich region (residues 125–135) N-terminal to a pair of retroviral-type CCHC zinc-binding domains [ZBD1 (residues 137–154) and ZBD2 (residues 160–176); (19)]. Site-specific substitutions of Lin28 are shown in red. (**B**) Primary and proposed secondary structures of TL-let-7g. Nonnatural nucleotides are shown in lowercase, residues of previously identified Lin28-binding sites in bold characters, substitution sites for 2-AP are as blue shadows, regions that were replaced by alternative structural elements are boxed, and Dicer cleavage sites are indicated by red dots.

RNA recognition by Lin28 is being intensively investigated given that it contains two highly conserved RNA-binding domains [Figure 1A; (41,50)]; an N-terminal cold-shock domain (CSD) and a C-terminal NCp7-like domain composed of one lysine-rich and arginine-rich (KR-rich) motif and two CCHC-type zinc-binding domains [ZBDs; (19)]. Recent crystal structures indicate that the Lin28 CSD binds with low sequence specificity to single-stranded RNAs derived from the terminal loop of pre-let-7 (TL-let-7) and fitting preferably either the 5′-NGNGAYNNN-3′ [Y = pyrimidine and N = any base; (51)] or the 5′-GUNNUNN-3′ (52) consensus. Crystal (51) and solution (53) structures also indicate that the NCp7-like domain specifically binds a G-rich region (5′-GGAG-3′) found at the 3′-end of TL-let-7. These structural data are consistent with the importance of the 5′-GGAG-3′ sequence for Lin28 binding and for the Lin28-dependent uridylation by the TUTase Zcchc11 in pre-let-7a-1 (17,52). In addition, biochemical studies have defined a G-rich bulge at the 5′-end of TL-let-7g (5′-UGAGGG-3′) as a primary binding site for Lin28 (18,19). In agreement with these results, binding studies using small single-stranded oligoribonucleotides and genome-wide studies of Lin28-associated mRNAs identified several G-rich and U-rich sequences as Lin28 targets (51,52,54–57), with a noted preference for G-rich sequences by the NCp7-like domain and for U-rich sequences by the CSD (51,57). Globally, these studies reveal the complexity of defining a simple consensus sequence for Lin28.

Although previous biochemical and structural studies have described three potential recognition sites for Lin28 on the terminal loop of pre-let-7 RNAs (Figure 1B), most studies report a 1:1 stoichiometry for the Lin28/TL-let-7 complex (15,17–19,51–53). Based on the observation of native gel supershifts in our previous Lin28-binding studies with TL-let-7g, we proposed that multiple molecules of Lin28 likely interact with pre-let-7g to fulfill its inhibitory function on let-7 biogenesis (19). More recently, such supershifts were also observed as a result of Lin28 binding to pre-let-7a-1 (55). In the present work, we characterize the mechanistic details for multimeric binding of Lin28 to pre-let-7 targets. We define three distinct Lin28 binding sites in the TL-let-7g and describe the stepwise assembly of three molecules of Lin28 to a single molecule of TL-let-7g. Furthermore, we define the respective roles of the CSD and NCp7-like domain of Lin28 in the formation of this multimeric complex and demonstrate its specificity towards terminal loops of the let-7 family of pre-miRNAs. Based on these results, we propose a model for stepwise assembly of Lin28 to the terminal loop of pre-let-7g and investigate how the processing enzyme Dicer intervenes in this process.

## MATERIALS AND METHODS

### RNA and protein preparation

All unlabelled RNAs longer than 14 nucleotides and all proteins used in this study are derived from murine sequences and were prepared as described previously (19), except for pre-let-7 RNAs used in the Dicer processing assay. Shorter RNAs and RNAs with fluorescent labels [5′-Cy3, 5′-Cy5 or internal 2-aminopurine (2-AP)] were obtained from Integrated DNA Technology (IDT). For the Dicer processing assay, the mono-uridylated human pre-let-7g (pre-let-7g-U) was purified from a CRISPR-pre-let-7g-U-ARiBo precursor (58), whereas the mono-uridylated human pre-let-7d (pre-let-7d-U) and pre-let-7a-1 (pre-let-7a-1-U) were purified from cis-cleavage of HH-pre-let-7-HDV precursors (59). For radiolabelling of RNAs, [5′-^32^P]-labelling and subsequent purification were performed as described previously (60). The unlabelled pre-let-7g-U, pre-let-7d-U and pre-let-7a-1-U RNAs used in the Dicer processing assay were phosphorylated and purified similarly using non-radioactive ATP.

### Determination of dissociation constants (*K*_d_) by electrophoretic mobility shift assay

For binding studies by electrophoretic mobility shift assay (EMSA), protein and ^32^P-labelled RNA samples were prepared in EMSA buffer (50 mM Tris pH 7.6, 50 mM NaCl, 10% glycerol, 0.05% NP-40 alternative and 2 mM DTT) and the binding reaction was performed as described previously (19). Binding reactions were loaded directly on a standard 8% native gel [37.5:1 polyacrylamide/bisacrylamide with Tris-Glycine buffer (25 mM Tris-Base and 200 mM glycine)] run at 200 V for 1 h with active water cooling at 4°C. The gels were then dried and exposed overnight on a storage phosphor screen (Bio-Rad). The ^32^P-labelled RNA was visualized with a Molecular Imager FX densitometer or a Personal Molecular Imager system (both from Bio-Rad), and the band intensities were quantified using ImageLab (version 4.1 from Bio-Rad was used throughout this study). The fraction of bound RNA was plotted against protein concentration and the data were fitted to the one-site binding equation or the Hill equation by non-linear regression analysis with OriginPro 8 (OriginLab).

### Stoichiometric binding assay by native gel electrophoresis

For the stoichiometric binding assay, 5-µM RNA samples (or 10× the final RNA concentration) were prepared by combining unlabelled RNA with 100 pM of ^32^P-labelled RNA, and these samples were heated and snap-cooled (heated at 95°C and snap-cooled on ice for 5 min) to promote hairpin formation. The protein samples were first diluted in EMSA buffer to the appropriate concentrations, and the binding reactions (20 µl) were initiated by adding 500 nM of RNA (or lower concentrations for results in Supplementary Figure S2A). For each stoichiometric binding assay, binding reactions were incubated at 4°C for 30 min and loaded directly on an 8% standard native gel run at 200 V for 2 h with active water cooling at 4°C. The gels were then dried, exposed and visualized as described for *K*_d_ determination. The fractions of RNA present in the bands of the 1:1, 1:2 and 1:3 complexes were quantified with ImageLab, and reported scores in Table 2 were obtained from at least two independent experiments.

### 2-Aminopurine fluorescence assay

Several TL-let-7g RNAs carrying a single adenine to 2-aminopurine (2-AP) substitution were used for the 2-AP fluorescence assay. Each 2-AP-modified RNA was first heated and snap-cooled to promote hairpin formation. The emission spectra of the 500-nM solution of 2-AP-modified RNA (or unmodified RNA used as a control) were recorded from 335/20 nm (335 nm with an emission slit of 20 nm) to 425/20 nm after excitation at 300/5 nm first in absence of protein and then 1 min after each protein addition from a concentrated stock. All experiments were performed with a Varian Cary Eclipse fluorimeter at 4°C in 50 mM Tris pH 7.6, 50 mM NaCl and 10% glycerol. Each difference emission spectrum was obtained by subtracting the emission spectrum of the sample containing the 2-AP-modified RNA with that containing unmodified RNA collected under the same protein concentration and buffer conditions. For each titration, the fluorescence emission intensity at 370 nm was normalized with respect to the highest fluorescence emission intensity observed at 370 nm for that specific titration experiment. The normalized fluorescence intensity at 370 nm was plotted as a function of protein concentration, and the data were fitted to the dose–response equation [y = A_1_ + (A_2_ − A_1_)/(1 + 10^((logx_0_ − x) × p)), where A_1_ is the bottom asymptote, A_2_ the top asymptote, x_0_ the EC_50_ and p the Hill slope] by non-linear regression analysis with OriginPro 8.

### Strand displacement assay monitored by fluorescence resonance energy transfer

Three different fluorophore-labelled (5′-Cy3 or 5′-Cy5) RNAs were used for the strand displacement assay by fluorescence resonance energy transfer (FRET). The forward strand (Cy5-FWD: 5′-Cy5-CGU ACA GAU UGA GGG UGA CAU CG-3′) was annealed to a perfectly complementary reverse strand (Cy3-REV_comp_: 5′-Cy3-CGA UGU CAC CCU CAA UCU GUA CG-3′) to form the complementary duplex (duplex_comp_) and to a partially complementary reverse strand (Cy3-REV_bulge_: 5′-Cy3-CGA UGU CAU AUC UGU ACG-3′) to form a duplex with a G-rich bulge (duplex_bulge_). Solutions containing 25 nM of both forward and reverse strands were heated and slow-cooled (heated 2 min at 95°C and slow-cooled at room temperature for 20 min) to promote duplex formation. The strand displacement was monitored by FRET on addition of a concentrated stock of protein to a solution of Cy3/Cy5-labelled RNA duplex in EMSA buffer. Following protein addition, the sample was equilibrated at 30°C for 1 min, and fluorescence emission data were recorded at 30°C on a Varian Cary Eclipse fluorimeter following an excitation of Cy3 at 535/20 nm. The Fret Index (F_Cy5_/F_Cy3_) was calculated from the emission of Cy5 at 670/10 nm (F_Cy5_) and Cy3 at 590/10 nm (F_Cy3_), and this Fret Index was normalized with respect to the Fret Index obtained in the absence of protein. The ΔFRET_max_ value corresponds to the maximum difference in normalized Fret Index. The normalized Fret Index was also plotted against total protein concentration and the data were fitted to the dose–response equation by non-linear regression analysis with OriginPro.

### Strand displacement assay monitored by native gels

This assay was performed with RNA duplexes formed by combining fluorophore-labelled RNAs (5′-Cy3 or 5′-Cy5) following by heating and slow cooling. As controls, individual fluorophore-labelled RNAs were also investigated, and those were prepared by heating and snap cooling. The protein binding reactions were performed at 25 nM or 250 nM RNA concentrations and separated on gels as described for the stoichiometric binding assay. The fluorophore-labelled RNAs were detected with the ChemiDoc MP system (Bio-Rad) set up for multiplex detection of Cy3 and Cy5 fluorophores.

### Dicer processing assay

Dicer cleavage reactions were performed as described for the stoichiometric binding assay but using a total volume of 20 µl containing 50 mM Tris pH 7.6, 250 mM NaCl, 2.5 mM MgCl_2_, 10% glycerol, 0.05% NP-40, 2 mM DTT, 500 nM unlabelled pre-miRNA, 40 pM ^32^P-labelled pre-miRNA, variable concentrations of Lin28 and 0.25 U of recombinant Dicer (Genlantis). These reactions were incubated 10 min on ice and then 1 h at 37°C. Afterwards, 0.05 U of proteinase K (Roche) was added; samples were incubated 15 min at 37°C; and the reactions were stopped by adding 40 µl of gel loading buffer (87% formamide, 25 mM EDTA, 0.02 % xylene cyanol, 0.02% bromophenol blue). Samples (15 µl) were loaded on a 10% polyacrylamide (19:1 polyacrylamide/bisacrylamide)/7 M urea gel, run at 500 V for 45 min and then dried, exposed and visualized as described for *K*_d_ determination. Band intensities for the full-length and cleaved pre-miRNA were quantified using ImageLab and used to derive the percentages of pre-miRNA cleavage. The relative Dicer processing efficiency is the percentage of pre-miRNA cleavage in the presence of Lin28 over that in the absence of Lin28.

### Error analysis

For *K*_d_ determination, 2-AP fluorescence and strand displacement assays, at least three independent binding experiments were performed. Reported values (*K*_d_, EC_50_, Fret Index, ΔFRET_max_) and their errors are respectively the average values and the standard deviations from the multiple experiments.

## RESULTS

### Major variants of the pre-let-7g terminal loop maintain high-affinity binding to Lin28

We previously determined by EMSA that Lin28 binds the terminal loop of pre-let-7g (TL-let-7g) with a very high affinity [*K*_d_ = 0.13 nM; (19)]. The binding data were best fitted using the Hill equation, with a Hill coefficient (*n*) of 2.9. Given that supershifts were also observed in our EMSA at high concentrations of Lin28, we hypothesized that multiple molecules of Lin28 could bind a single molecule of TL-let-7g (19). To better understand the determinants of TL-let-7g binding to Lin28, we performed our EMSA with three variants of TL-let-7g (Figure 1B) in which key secondary structure elements were modified, namely the 5′ G-rich bulge (Δbulge), the internal loop (Δiloop) and the hairpin loop (GNRA loop). Remarkably, these variations do not greatly affect the binding affinity of Lin28 to TL-let-7g (Table 1), as the averaged *K*_d_ values increase by no more than 7-fold relative to the wild-type sequence. The largest effect is observed with the Δbulge variant (*K*_d_ of 0.9 nM) previously shown to abolish the TL-let-7g binding of a variant of Lin28 containing only the NCp7-like domain [Lin28_119__–180_; (19)]. The lack of a similar effect with the full-length protein concur with the hypothesis that Lin28 has the ability to bind multiple sites on TL-let-7g and that the variants being studied may destroy and/or conceal one or more of the Lin28 binding sites, but still allow high-affinity Lin28 binding to other sites.

**Table 1.** Dissociation constants (*K*_d_)^a^ for binding of Lin28 to RNAs derived from TL-let-7g^a^

RNA	Sequences (5′ to 3′)^b^	*K*_d_ (nM)
TL-let-7g	GCAGAUU**GAGGG**UCU**AUGAUAC**CACCCGGUACA**GGAG**AUAUCUGCA	0.13 ± 0.02
		*n* = 2.9 ± 0.7^c^
TL-let-7g Δbulge	GCAGAU––-**G**UCU**AUGAUAC**CACCCGGUACA**GGAG**AUAUCUGCA	0.9 ± 0.2
		*n* = 2 ± 1
TL-let-7g Δiloop	GCAGAUU**GAGGG**UCU-**UG**-**UAC**CACCCGGUACA–**AG**AUAUCUGCA	0.52 ± 0.09
		*n* = 1.3 ± 0.1
TL-let-7g GNRA loop	GCAGAUU**GAGGG**UCU**AUGAUAC**CGCAAGGUACA**GGAG**AUAUCUGCA	0.4 ± 0.2
		*n* = 1.46 ± 0.01
TL5-18	AUU**GAGGG**UCUAUA	1.7 ± 0.4
TL5-18V	AUU**GACGC**UCUAUA	>5000
TL13-26	UCU**AUGAUAC**CACC	25–75^d^
TL13-26V	UCU**AUCAAAC**CACC	n.b.^e^
TL19-32	** AUAC**CACCCGGUAC	n.b.^e^
TL28-41	GGUACA**GGAG**AUAU	0.29 ± 0.08
TL28-41V	GGUACA**CCAC**AUAU	25–75^d^
TL33-46	A**GGAG**AUAUCUGCA	0.5 ± 0.1

^a^Each *K*_d_ value and its associated error are the average and standard deviation, respectively, from at least three independent experiments.

^b^Residues in bold are part of previously identified Lin28 binding sites (see text).

^c^The *K*_d_ value was obtained from a previous study using the Hill equation (19).

^d^Only an approximate value could be obtained in these cases.

^e^No specific binding observed. The gel mobility shift assays display smearing, indicating nonspecific binding.

### The terminal loop of pre-let-7g comprises three distinct binding sites for Lin28

To precisely identify the different binding sites of Lin28 on TL-let-7g, we performed additional binding assays by EMSA using 14-nt RNA fragments derived from the TL-let-7g. These short RNAs were designed to cover the entire terminal loop of pre-let-7g with partial overlaps and are named according to the matching segment of residues within TL-let-7g (Table 1 and Figure 1B). For example, TL5-18 containing the sequence of the 5′ G-rich bulge of TL-let-7g, residues 5 to 18, was investigated for Lin28 binding by EMSA (Supplementary Figure S1A). The plot of the bound RNA fraction versus Lin28 protein concentration fits the one-site binding equation (Supplementary Figure S1B) to yield a *K*_d_ of 1.7 nM (Table 1). Binding of Lin28 to a shorter fragment derived from TL5-18 (TL6-13) was not detectable at protein concentrations up to 5 µM by EMSA (data not shown), suggesting that a minimal RNA fragment length, between 9-14 nt, is necessary for high-affinity binding. Amongst the five short RNAs with native sequences tested (TL5-18, TL13-26, TL19-32, TL28-41, TL33-36), only one (TL19-32) did not bind Lin28 by EMSA (Table 1). In addition to TL5-18, subnanomolar binding affinity is observed for TL28-41 (*K*_d_ = 0.29 nM) and TL33-46 (*K*_d_ = 0.5 nM), whereas nanomolar binding affinity is observed for TL13-26 (25–75 nM). The binding data with these four RNAs could be fitted to the one site binding equation and only a single shifted band is observed by EMSA, indicating that these short RNAs contain a single binding site for Lin28.

Interestingly, these four short RNAs that bind Lin28 with high affinity contain previously identified binding sites for Lin28 (in bold in Table 1). First, the TL5-18 RNA contains the 5′ G-rich bulge recently identified as a primary binding site for Lin28 and its NCp7-like domain in pre-let-7g (18,19). Also, both TL28-41 and TL33-46 contain the 3′-GGAG sequence defined as a key determinant of Lin28 binding and function targeted specifically by the NCp7-like domain (16,17,51–53). To confirm that the two G-rich sequences in these short RNAs were important for the observed high-affinity binding, we tested the binding of short RNA variants with nucleotide changes of key G residues (17–19,51–53). As expected, replacement of key guanines in TL5-18 and TL28-41, either abolished (TL5-18V) or greatly reduced (TL28-41V) Lin28 binding (Table 1). Despite the importance of these G residues, no binding could be observed in our assay between the NCp7-like domain (Lin28_119__–__180_) and the G-rich fragments (TL5-18, TL28-41 or TL33-46; data not shown), indicating that the CSD is required for binding these short RNAs. Finally, TL13-26 contains the previously identified AUGAUAC sequence recognized by the CSD of Lin28 (51,52). Again, nucleotide changes within TL13-26 at key positions for CSD recognition (51) prevent binding to Lin28 (Table 1; TL13-26V). In summary, by using short and most likely single-stranded RNAs, three distinct Lin28 binding sites were identified on TL-let-7g. Because most of these sites do not adopt a single-stranded conformation in the context of TL-let-7g (18,19), it is not clear yet if they all are accessible to allow binding of multiple molecules of Lin28 to a single molecule of TL-let-7g.

### Stepwise assembly of Lin28 on the terminal loop of pre-let-7g yields a stable 1:3 complex

**Figure 2.**
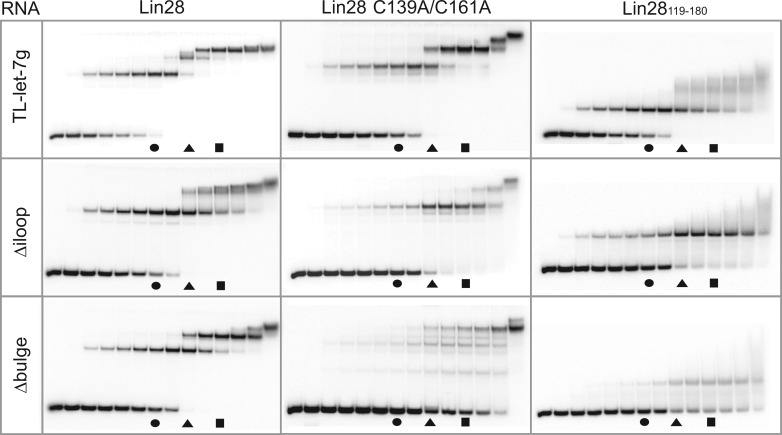
Stoichiometric binding assay by native gel electrophoresis for Lin28 binding to TL-let-7g RNA and effect of select RNA and protein variants on the stoichiometry of the complex. Each assay is performed with 0.5 µM RNA, including 10 pM 5′-[^32^P]-labelled RNA, and increasing concentrations of protein (0.00, 0.05, 0.25, 0.375, 0.50, 0.625, 0.75, 1.00, 1.25, 1.50, 1.75, 2.00, 2.50 and 5.00 µM). The gel lanes with RNA:protein ratios of 1:1.5, 1:2.5 and 1:3.5 are identified by a circle, a triangle and a square, respectively.

To determine if multiple molecules of Lin28 bind one molecule of TL-let-7g, we optimized a stoichiometric binding assay. In this assay, an increasing amount of protein (0.1× to 10× the RNA concentration) is added to a non-negligible quantity (500 nM) of RNA. If multiple molecules of Lin28 bind TL-let-7g in a non-concerted fashion, supershifts will be observed on a native gel. In fact, the stoichiometric binding assay of Lin28 to TL-let-7g (Figure 2, top left panel) results in the sequential appearance of a band shift, a supershift and a super-supershift that are almost fully populated at RNA:protein ratios of 1:1.5, 1:2.5 and 1:3.5, respectively. The mobility of the free RNA band and that of the first shift are invariable at RNA concentrations ranging from 1 pM to 0.5 µM (data not shown), which strongly indicates that TL-let-7g forms a monomeric hairpin in its free and bound forms. Furthermore, 1:1 binding was previously observed for similar complexes by sedimentation equilibration ultracentrifugation (51), size-exclusion chromatography and mass spectrometry (61) at equimolar concentrations of RNA and protein. Thus, the observed shifts most likely correspond to RNA:protein stoichiometries of 1:1, 1:2 and 1:3. Additional molecules of Lin28 may associate transiently to the 1:3 complex, as indicated by smearing of the 1:3 complex band at the highest RNA:protein ratios (1:5 and 1:10). Remarkably, the largest stable multimeric complex (designated the 1:3 complex) is assembled in a stepwise manner; the free RNA must be completely shifted in a 1:1 complex, prior to formation of the 1:2 and 1:3 complexes.

This stoichiometric binding assay was also performed at lower concentration of TL-let-7g (25, 50 and 250 nM; Supplementary Figure S2A). The 1:1, 1:2 and 1:3 complexes form in all cases, but higher protein:RNA ratios are needed as the RNA concentration is reduced. Formation of stable multimeric complexes was also tested with the parental pre-let-7g at 500 nM RNA (Supplementary Figure S2B). Compared with results with TL-let-7g, somewhat higher protein:RNA ratios are needed for formation of the 1:1 and 1:2 and 1:3 complexes, and the latter is more diffuse, possibly due to the stabilizing effect of the longer stem of pre-let-7g or to its contribution to non-specific Lin28 binding.

### Both RNA-binding domains of Lin28 contribute to its stepwise assembly on TL-let-7g

To examine the individual roles of the CSD and NCp7-like domain of Lin28 in the assembly of the 1:3 complex, stoichiometric binding assays were repeated with Lin28 variants containing only a single functional RNA domain (Figures 1A and 2 and Table 2). The Lin28_119__–__180_ variant, which only contains the NCp7-like domain known to target the 5′ G-rich bulge (19), forms a 1:1 complex with TL-let-7g (Figure 2, top right panel) with an affinity similar to the full-length protein, but forms 1:2 and 1:3 complexes with TL-let-7g very inefficiently and in a non-orderly fashion. In contrast, the Lin28 C139A/C161A variant, which contains a functional CSD but a dysfunctional NCp7-like domain, can assemble to form 1:1, 1:2 and 1:3 complexes (Figure 2, top middle panel), but at higher protein concentrations than for the wild-type Lin28, such that it prevents formation of the 1:3 complex at an RNA:protein ratio of 1:3.5 (Table 2). Given that the binding affinity for TL-let-7g of the NCp-7 like domain (*K*_d_ = 1.3 nM) is higher than for the CSD [*K*_d_ = 126 nM; (19)], it is not surprising to find in our stoichiometric assay that Lin28 C139A/C161A is slightly more deficient at forming the 1:1 complex than the NCp7-like domain (see Table 2). Thus, the NCp7-like domain is important for formation of a high-affinity 1:1 complex and it facilitates assembly of 1:2 and 1:3 complexes, but the intrinsic ability of Lin28 to efficiently multimerize on TL-let-7g is imparted by the CSD.

**Table 2.** Ability of Lin28 and variants to form multimeric complexes with various RNAs^a^

RNA	Lin28			Lin28 C139A/C161A			Lin28_119–180_		
	1:1	1:2	1:3	1:1	1:2	1:3	1:1	1:2	1:3
TL-let-7g	++++	++++	++++	++	++	−	+++	+	−
TL-let-7g Δiloop	+++	+/++	++	+	−	−	++	−	−
TL-let-7g Δbulge	++	++	−	−/+	+	−	−/+	−	−
TL-let-7a-1	++++	+++	++	+	−	−	+++	−	−
TL-let-7d	++++	++	+/++	+++	−/+	−	+++	+/++	−
TL-miR-99b	+++	−/+	−	+	−	−	−	−	−

^a^The score indicated the amount of RNA present in the indicated stoichiometric ratio: ++++, >75%; +++, 50–75%; ++, 25–50%; +, 5–25%; −, 0–5%. The ability of the protein to form a 1:1, 1:2 or 1:3 complex with the RNA was respectively quantified at 0.750, 1.250 and 1.750 µM of protein from gels shown in Figures 2–3.

Interestingly, stoichiometric binding of one or two equivalents of Lin28 C139A/C161A to TL-let-7g is not affected by the binding of one equivalent of Lin28_119__–180_ (Supplementary Figure S3), but rather lead to formation of 1:1:1 and 1:1:2 RNA:Lin28_119__–180_:Lin28 C139A/C161A complexes as indicated by the gel mobility of the complexes. Thus, the Lin28_119__–180_ and Lin28 C139A/C161A variants target different residues in their respective 1:1 complex, in agreement with the different sequence preference for the NCp7-like domain and the CSD (51,57).

We also investigated stoichiometric binding to two TL-let-7g variants [Figure 1B; (19)]. The TL-let-7g Δiloop variant was previously shown to have a minor effect on binding of Lin28_119__–__180_, in agreement with the fact that Lin28_119__–__180_ mainly recognizes the 5′ G-rich bulge (19). Similarly, this variant reduces to a limited extent the ability of Lin28_119__–__180_ to form a 1:1 complex (Figure 2 and Table 2). In addition, it reduces the ability of both Lin28 and the Lin28 C139A/C161A variant to form 1:1, 1:2 and 1:3 complexes (Figure 2 and Table 2). Given that both the 5′-AUGAUAC-3′ and 5′-GGAG-3′ binding sites are destroyed in the Δiloop variant, the limited formation of the 1:2 and 1:3 complexes with these proteins likely results from the low specificity of the CSD (52). Deletion of the 5′ G-rich bulge (Δbulge; Figure 1B), previously identified as the main binding site for Lin28_119__–__180_ (19), has a larger effect on stoichiometric binding. As expected, the Δbulge variant strongly reduces the ability of Lin28_119__–__180_ to form a 1:1 complex (Figure 2, bottom panels and Table 2). In addition, the Δbulge variant significantly reduces the ability of Lin28 and Lin28 C139A/C161A to form 1:1, 1:2 and 1:3 complexes. Lin28 forms 1:1 and 1:2 complexes with the Δbulge variant, but not the 1:3 complex at an RNA:protein ratio of 1:3.5 (Table 2), in agreement with the loss of a high-affinity binding site on the RNA. Comparatively, Lin28 C139A/C161A is more deficient than Lin28 at forming 1:1 and 1:2 complexes with the Δbulge variant and even loses its ability for stepwise assembly. These results support the idea that the NCp7-like domain and the 5′ G-rich bulge both play an important role in initiating the ordered assembly of the multimeric complexes, as previously suggested (19).

### Lin28 specifically forms stable 1:3 complexes with terminal loops of other miRNA precursors from the let-7 family

**Figure 3.**
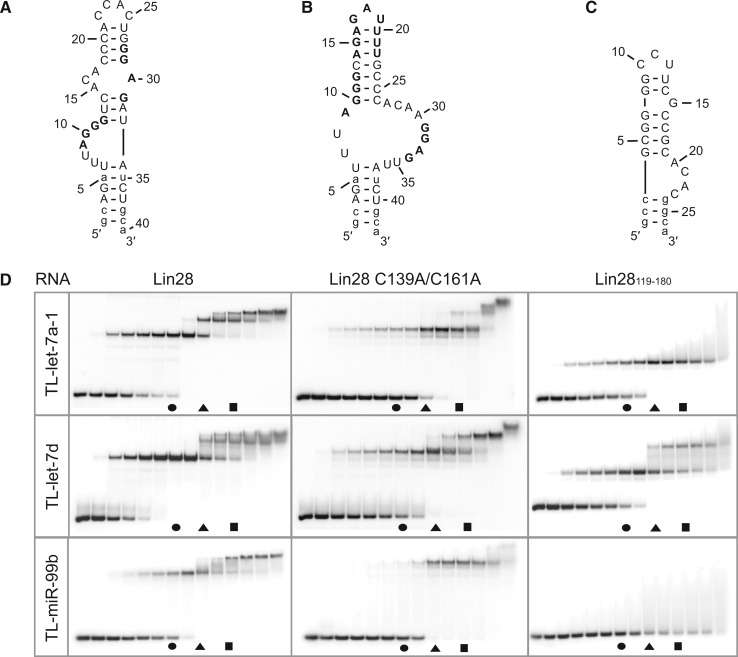
Stoichiometric binding assay by native gel electrophoresis for Lin28 binding to other pre-let-7 terminal loops. Sequences and proposed secondary structures of (**A**) TL-let-7a-1, (**B**) TL-let-7d and (**C**) TL-miR-99b with bold residues representing previously identified or predicted Lin28-binding sites (see text). Nonnatural nucleotides are shown in lowercase. (**D**) Stoichiometric binding assay of TL-let-7a-1, TL-let-7d and TL-miR-99b with Lin28, Lin28 C139A/C161A and Lin28_119–180_. Each assay is performed with 0.5 µM RNA, including 10 pM 5′-[^32^P]-labelled RNA, and increasing concentrations of protein (0.00, 0.05, 0.25, 0.375, 0.50, 0.625, 0.75, 1.00, 1.25, 1.50, 1.75, 2.00, 2.50 and 5.00 µM). The gel lanes with RNA:protein ratios of 1:1.5, 1:2.5 and 1:3.5 are identified by a circle, a triangle and a square, respectively.

Stoichiometric binding was also investigated between Lin28 and the terminal loops of other miRNA precursors to determine if, like TL-let-7g, these terminal loops have the ability for stepwise assembly of a stable 1:3 complex. Interestingly, both TL-let-7a-1 and TL-let-7d can form 1:1, 1:2 and 1:3 complexes, although the stepwise assembly of the 1:2 and 1:3 complexes requires higher Lin28 concentration than for TL-let-7g (Figure 3 and Table 2). Both TL-let-7a-1 and TL-let-7d contain 5′ and 3′ G-rich sequences (in bold in Figure 3AB), and TL-let-7d also contains an established CSD binding site [the 5′-AGAGAUUUU-3′ sequence in bold in Figure 3B; (51,52)]. For TL-let-7d, the highly accessible 5′-GGAG-3′ binding site and the partially accessible 5′-AGAGAUUUU-3′ site likely contribute to formation of a high-affinity 1:1 complex, as observed in the X-ray crystal structure [Figure 3D; (51,52)]. Binding to Lin28 variants is similar for TL-let-7a-1 and TL-let-7d compared with TL-let-7g, with the Lin28_119__–__180_ variant binding with high affinity to at least one site and the Lin28 C139A/C161A variant allowing stepwise assembly of multimeric complexes (Figures 2 and 3), but at higher protein concentration than Lin28.

To further examine the specificity for stepwise assembly of multimeric complexes between Lin28 and TL-let-7 RNAs, the binding of Lin28 was also investigated with the terminal loops of miR-99b (TL-miR-99b, Figure 3C) and miR-21a (TL-miR-21a, Supplementary Figure S4A). It was previously demonstrated that the biogenesis of miR-99b and miR-21a is unaffected by the expression or the silencing of Lin28 (15,16,24). In agreement with these results, we measured *K*_d_ values of 15 nM for TL-miR-99b and 13 nM for TL-miR-21a for formation of a 1:1 complex with Lin28 (data not shown), which represent two orders of magnitude weaker binding than for TL-let-7g [*K*_d_ = 0.13 nM; (19)]. Furthermore, in our stoichiometric binding assay Lin28 has a reduced ability to form 1:1 and 1:2 complexes with TL-miR-99b compared with TL-let-7g and does not form a 1:3 complex under the conditions being investigated (Figure 3D). Similar results were obtained with TL-miR-21a (Supplementary Figure S4B). Furthermore, stoichiometric binding of the Lin28_119__–__180_ and Lin28 C139A/C161A variants is severely reduced with TL-miR-99b compared with TL-let-7g (Figure 3D). Taken together, these results indicate that stepwise assembly is markedly impaired with TL-miR-99b and TL-miR-21a relative to TL-let-7 RNAs.

### Dynamic assembly of Lin28 on the terminal loop of pre-let-7g

**Figure 4.**
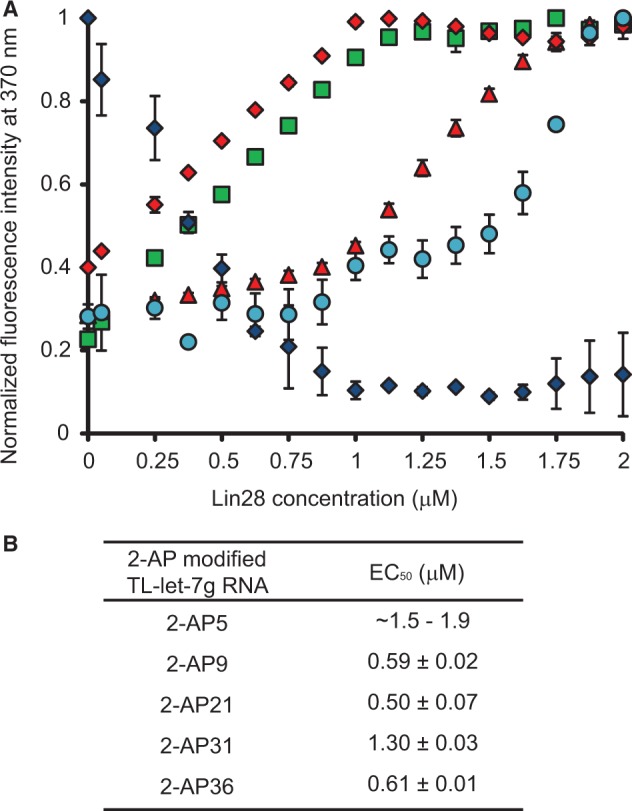
Effect of Lin28 on the fluorescence intensity of TL-let-7g containing individual 2-AP modifications. (**A**) Normalized fluorescence intensity at 370 nm of 2-AP5 (cyan dots), 2-AP9 (green squares), 2-AP21 (blue diamonds), 2-AP31 (red triangles) and 2-AP36 (red diamonds) as a function of Lin28 concentration. Each titration point and the associated error bars are respectively the average and standard deviation from multiple experiments. (**B**) Reported EC_50_ values and their errors obtained from data in (A) by fitting the normalized fluorescence intensity at 370 nm with respect to Lin28 concentration using the dose–response equation.

To better understand the dynamic assembly of Lin28 on TL-let-7g RNA, we carried out a fluorescence assay using a series of TL-let-7g RNAs containing single adenine to 2-aminopurine (2-AP) modifications (2-AP5, 2-AP9, 2-AP21, 2-AP31 and 2-AP36; Figure 1B). The fluorescence emission of 2-AP is highly sensitive to the immediate environment of the base analogue, particularly stacking interactions that can substantially quench the high quantum yield of free 2-AP (62–66). Therefore, 2-AP is an ideal reporter for changes in RNA structure and dynamic resulting from protein binding. Here, the fluorescence emission spectra of the modified TL-let-7g were first collected in the absence of proteins using the same detection parameters (Supplementary Figure S5A). The low fluorescence of 2-AP5 is consistent with this residue being part of a stable stem, whereas the higher fluorescence of the other 2-AP-modified TL-let-7g RNAs indicates that these modified residues (A9, A21, A31 and A36) are part of more flexible regions in the RNA. Following the addition of Lin28, 2-AP21 undergoes a significant decrease in fluorescence intensity with a measured EC_50_ of 0.50 µM [Figure 4 (blue diamonds) and Supplementary Figure S5D]. This result is consistent with stabilization of 2-AP stacking, most likely as a result of direct binding with Lin28, as A21 is located in a previously identified CSD binding site (5′-AUGAU**A**C-3′). According to the crystal structure of a complex between derivatives of Lin28 and TL-let-7g, A21 should stack between Trp46 and C22 when bound to the CSD [pdb code:3TS2; (51)]. The other 2-AP-modified TL-let-7g RNAs undergo fluorescence increases on addition of Lin28 [Figure 4 and Supplementary Figure S5], either through a direct interaction with Lin28 or through destabilization of the RNA structure. Given that the EC_50_ values of 0.5–0.6 µM for 2-AP9, 2-AP21 and 2-AP36 nearly match the point of half binding for the first shift observed with the stoichiometric binding assay (Figure 2), these results indicate that binding of the first protein affects the local environment of multiple nucleotides of TL-let-7g. Furthermore, the EC_50_ of 1.3 µM for 2-AP31 [Figure 4 (red triangles) and Supplementary Figure S5E] is consistent with binding of a second molecule of Lin28 on TL-let-7g. Only a small increase in fluorescence intensity was observed for 2-AP5 at high Lin28 concentrations, indicating that the A5-U41 base pair of TL-let-7g remains stable until the formation of the 1:3 complex [Figure 4 (cyan dots) and Supplementary Figure S5B]. In summary, formation of the 1:1 complex significantly remodels the internal loop by affecting residues within the three Lin28 binding sites, whereas formation of the 1:2 and 1:3 complexes appear to have more localized effects on individual Lin28-binding sites.

### The CSD is responsible for the RNA melting activity of Lin28

**Figure 5.**
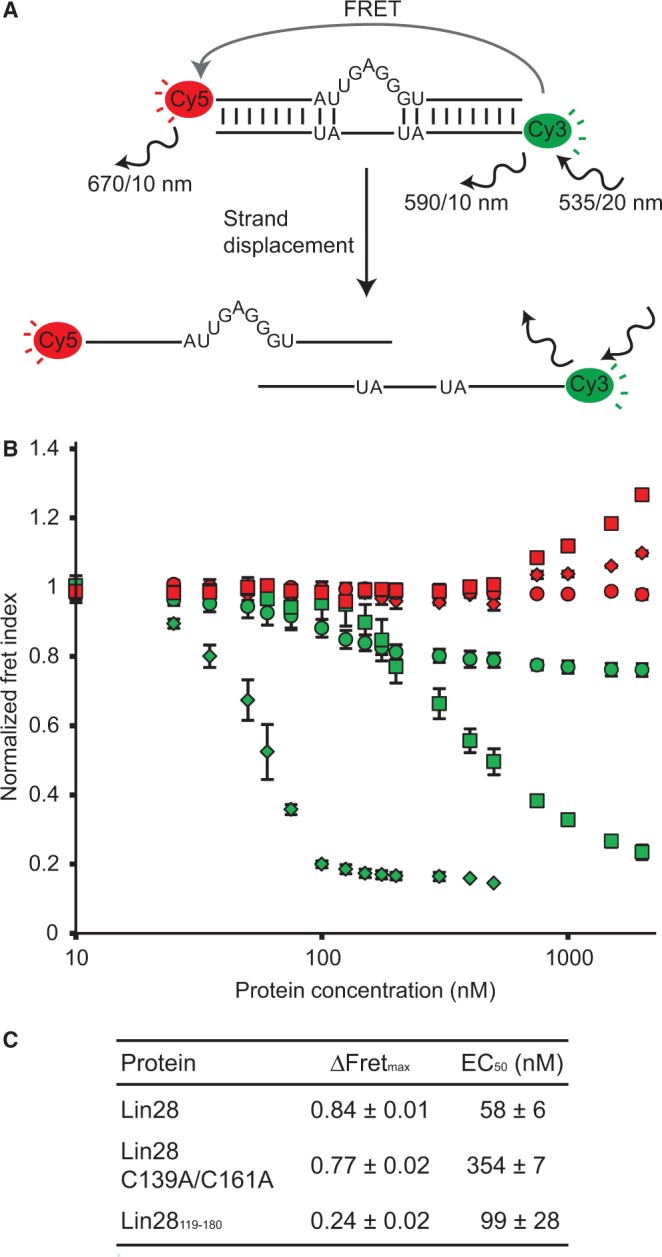
Strand displacement assay monitored by FRET to test the RNA melting activity of Lin28. (**A**) Schematic of the RNA melting activity of Lin28 on the duplex_bulge_ RNA, a Cy3/Cy5-labelled duplex. Relevant excitation and emission frequencies are indicated with associated slit widths. (**B**) Normalized Fret Index (F_Cy5_/F_Cy3_) of Cy3/Cy5-labelled RNA duplexes (25 nM) as a function of total protein concentration (10, 25, 35, 50, 60, 75, 100, 125, 150, 175, 200, 300, 400, 500, 750, 1000, 1500 and 2000 nM). Proteins were sequentially added to both the duplex_bulge_ (Lin28, green diamonds; Lin28 C139A/C161A, green squares; and Lin28_119–180_, green circles) and the control duplex_comp_ (Lin28, red diamonds; Lin28 C139A/C161A, red squares; and Lin28_119-180_, red circles). (**C**) Values of ΔFRET_max_ and EC_50_ derived from results shown in (B).

To investigate if Lin28 carries an RNA melting activity responsible for destabilizing the TL-let-7g on formation of the 1:1 complex, we adapted an established fluorescence-based strand displacement assay [Figure 5A; (67)]. This assay relies on determining the FRET index (F_Cy5_/F_Cy3_) following Cy3 excitation for an RNA duplex composed of 5′-Cy5-labelled and 5′-Cy3-labelled strands. The fluorescence emission of Cy5 is strongly dependent on the annealing of the two strands, such that the decrease in fluorescence intensity on addition of protein infers RNA melting activity. Here, two different RNA duplexes were used; one contains a G-rich bulge mimicking the 5′-bulge of TL-let-7g (duplex_bulge_) and the other is the equivalent duplex without the bulge such that the two strands are perfectly complementary (duplex_comp_).

The strand displacement assay performed with 25 nM duplex_bulge_ reveals the RNA melting activity of both Lin28 and the Lin28 C139A/C161A variants, with respective ΔFRET_max_ of 0.84 and 0.77 and EC_50_ of 58 nM and 354 nM (Figure 5B and C). The fluorescence emission profiles clearly demonstrate that the decrease in FRET index on addition of Lin28 is caused by an emission increase from Cy3 combined with an emission decrease from Cy5, which is consistent with the separation of the two fluorophores (Supplementary Figure S6AC). Furthermore, the stoichiometric binding assay performed with duplex_bulge_ at 25 and 250 nM provides further evidence for dissociation of the duplex_bulge_ in two separate strands as a result of addition of Lin28 (Supplementary Figures S7 and S8). For the Lin28_119__–__180_ variant, the strand displacement assay yields a smaller ΔFRET_max_ value of 0.24 with an EC_50_ of 99 nM, which is indicative of a specific binding event at the bulge that moves the fluorophores further apart relative to each other without displacing the strands, although strand displacement may occur to a limited extent (Figure 5 and Supplementary Figures S6E, S7E and S8E). Thus, the RNA melting activity of Lin28 is present at higher protein concentration for the Lin28 C139A/C161A variant, but essentially absent for the Lin28_119__–__180_ variant under all tested conditions. In contrast, Lin28 and its derivatives do not melt the perfectly complementary RNA duplex (duplex_comp_), as no decrease in FRET index was observed (Figure 5 and Supplementary Figure S6), indicating that the G-rich bulge is essential for this RNA melting activity. In summary, the strand displacement assay demonstrates that the RNA melting activity of Lin28 belongs predominantly to its CSD, but is enhanced by the NCp7-like domain of Lin28 due to its high-affinity binding to the G-rich bulge.

### Formation of the 1:1 complex is not always sufficient for maximum inhibition of Dicer cleavage in vitro

**Figure 6.**
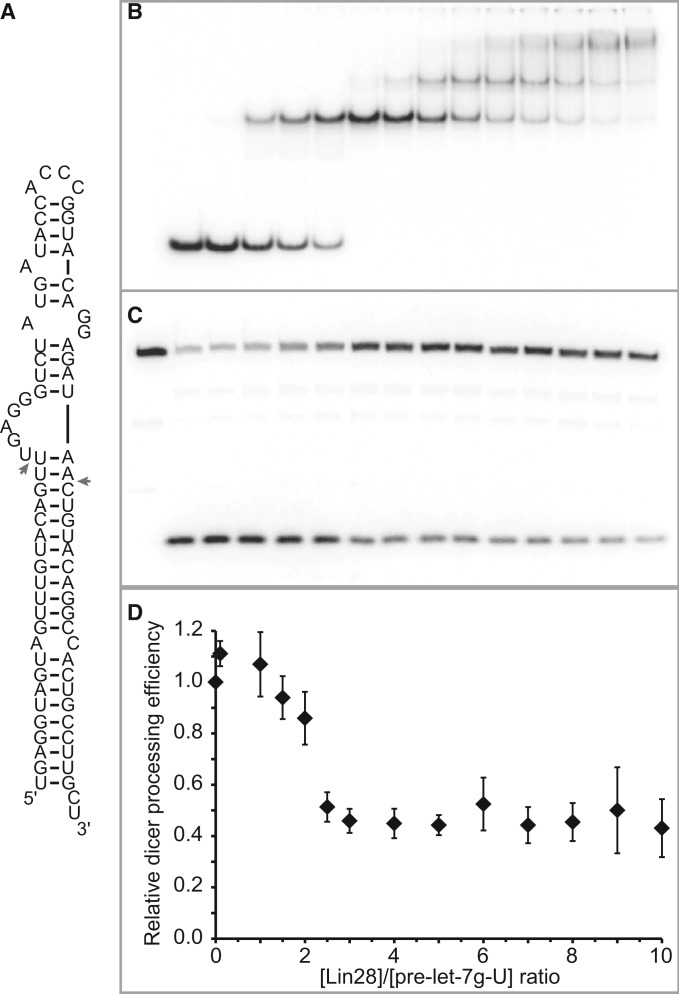
Dicer processing assay of pre-let-7g-U. (**A**) Primary and proposed secondary structures of the pre-let-7g-U RNA. Dicer cleavage sites are identified with arrows. (**B**) Stoichiometric binding assay detected by native gel electrophoresis for Lin28 binding to pre-let-7g-U. Each assay is performed with 0.5 µM 5′-phosphorylated RNA, including 40 pM 5′-[^32^P]-labelled RNA and increasing concentration of Lin28 (0.00, 0.05, 0.50, 0.75, 1.25, 1.50, 2.00, 2.50, 3.00, 3.50, 4.00, 4.50 and 5.00 µM). (**C**) Dicer processing assay detected by denaturing gel electrophoresis of pre-let-7g-U under varying Lin28 concentrations. The first well contains 0.5 µM RNA without Lin28 and Dicer. The subsequent wells are for the Dicer assay performed under the same conditions as for the stoichiometric binding assay (in B), but with an additional 0.25 U of Dicer enzyme. (**D**) Relative Dicer processing efficiency plotted against Lin28/pre-let-7g-U concentration ratios (*n* = 2).

Lin28 is known to inhibit the cleavage activity of Dicer on let-7 precursors, but it is not clear where this inhibition takes place in the stepwise assembly of Lin28 on pre-let-7g. To clarify this, we tested the effect of Lin28 concentration on Dicer cleavage of pre-let-7g-U, an optimal Dicer substrate containing a 5′-phosphate (68) and mono-uridylated at its 3′-end [Figure 6A; (30)]. In parallel, we performed a stoichiometric binding assay with pre-let-7g-U under the same high salt conditions as those used for Dicer cleavage (Figure 6B). A 60% reduction of Dicer cleavage (from ∼75% to ∼32% cleavage) is observed at Lin28 concentrations that allow formation of the 1:1 complex (Figure 6C and D). However, further increase in Lin28 concentration to allow formation of the 1:2 and 1:3 complexes do not further reduce Dicer cleavage. These results indicate that binding of one molecule of Lin28 to pre-let-7g-U is sufficient to significantly inhibit Dicer cleavage and that this level of inhibition is maintained on binding of additional molecules of Lin28.

Similar Dicer processing assays were performed with pre-let-7d-U and pre-let-7a-1-U, two additional Dicer substrates containing a 5′-phosphate (68) and mono-uridylated at their 3′-end [Supplementary Figures S9A and S10A; (30)]. Similarly to pre-let-7g-U, a 70% reduction of Dicer cleavage (from ∼91% to ∼24% cleavage) is observed for pre-let-7d-U at a Lin28 concentration that allows for formation of the 1:1 complex, and further increases in Lin28 concentration that allow for formation of the 1:2 and 1:3 complexes do not further reduce Dicer cleavage (Supplementary Figure S9C and D). In contrast, Dicer cleavage of pre-let-7a-1-U is reduced by 10% at a Lin28 concentration that allows for formation of the 1:1 complex, and is further reduced by 35% and 45% with Lin28 concentrations that allow for formation of the 1:2 and 1:3 complexes, respectively. Thus, although binding of one molecule of Lin28 to pre-let-7g-U and pre-let-7d-U is sufficient for maximum Dicer cleavage, multimerization of Lin28 is required for maximum Dicer inhibition with pre-let-7a-1-U as the substrate.

## DISCUSSION

In this work, we elucidate the molecular mechanism by which Lin28 interacts with the terminal loop of pre-let-7g. This binding mechanism, which involves stepwise assembly of three molecules of Lin28 on the pre-miRNA terminal loop, is detailed below in regard to an assembly model that recapitulates results presented here and helps clarify what was thought to be conflicting data associated with previous studies. In addition, we examine the significance of this stepwise assembly for in vivo regulation of pre-let-7 biogenesis.

### Binding of Lin28 to short RNAs

Using short 14-nt RNAs derived from TL-let-7g, we identified three distinct Lin28 binding sites on TL-let-7g. Fragments containing a conserved G-rich sequence bind with very high affinity to Lin28, those containing the 5′-GGAG-3′ motif binding with somewhat higher affinity (*K*_d_ of 0.3–0.5 nM) than those containing the 5′-GAGGG-3′ motif (*K*_d_ of 1.7 nM). These binding data are in agreement with previous studies in which short conserved G-rich elements located at both the 5′-end [5′-UGAGGG-3′; (18,19)] and the 3′-end [5′-GGAG-3′; (17,52)] of the terminal loop of pre-let-7g were individually defined as the main determinants of Lin28 binding. Short G-rich elements are also predominantly found in target sequences of Lin28-associated mRNAs as part of genome-wide studies (54–57,69). In our study with 14-nt RNA fragments, mutations of key G residues abolished high-affinity binding to Lin28, as expected for recognition by the NCp7-like domain. High-affinity binding was lost when we replaced the 14-nt TL5-18 fragment by an 8-nt 5′-UGAGGGUC-3′. Surprisingly, high-affinity binding to 14-nt G-rich fragments was also lost when we used a Lin28 variant containing just the NCp7-like domain, even though the NCp7-like domain recognizes the G-rich bulge with high affinity as part of TL-let-7g [*K*_d_ = 1.3 nM; (19)]. Binding of the NCp7-like domain to TL-let-7g may be favoured by the presence of nearby helical elements or the unique structure of the bulge. In agreement with our results, low affinity binding (*K*_d_ = 0.4 µM) was observed between an isolated NCp7-like domain and a G-rich RNA heptamer by isothermal titration calorimetry (53). Clearly, high-affinity binding of Lin28 to these 14-nt fragments is not limited to the NCp7-like domain, but likely involves the CSD, which is known to bind short RNAs (6–9 nt) with low-sequence specificity (52). Thus, both the NCp7-like domain and the CSD contribute to recognition of these G-rich sites by Lin28, and we defined a minimum length between 9 and 14 nt for high-affinity binding to G-rich fragments derived from pre-let-7g.

The TL13-26 fragment containing the 5′-AUGAUAC-3′ region from TL-let-7g binds Lin28 with high affinity (*K*_d_ of 25–75 nM). The 5′-AUGAUAC-3′ sequence directly interacts with the CSD in a crystal structure of a complex formed by Lin28 and pre-let-7g derivatives (51), and its importance for CSD binding is supported here by the loss of binding observed with the TL13-26 variant containing the 5′-AU**C**A**A**AC-3′ mutation. These results are in agreement with the ability of the CSD of Lin28B from *Xenopus tropicalis* (*Xtr*) to bind RNA fragments of 6-9 nts derived from the *Xtr*-pre-let-7 terminal loop with low nanomolar affinity (52). Given this observation and the fact that TL13-26 does not contain a GNG or GNNG sequence (19,53), binding to this fragment may only involve the CSD of Lin28.

### Model for stepwise assembly of Lin28 on pre-let-7g

**Figure 7.**
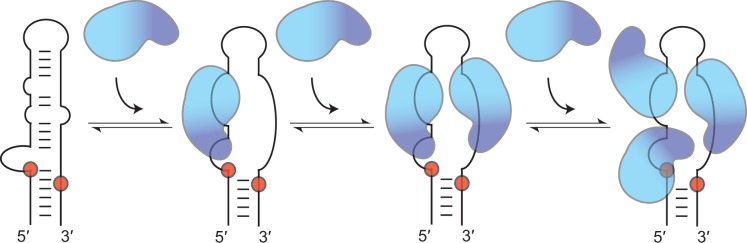
Schematic representation of the proposed model for the stepwise assembly of Lin28 on the terminal loop of pre-let-7g. In this model, only the RNA-binding domains of Lin28 are represented, with the NCp7-like domain in dark blue and the CSD in cyan. The first molecule of Lin28 initiates complex formation through interactions between its NCp7-like domain and the conserved 5′-GAGGG-3′ site at the 5′-bulge and between its CSD and the nearby 5′-AUGAUAC-3′ site. These interactions on the 5′-strand destabilize Watson–Crick base pairs within the terminal loop and exposes the 3′-strand, making it available for binding a second molecule of Lin28, with its NCp7-like domain targeting the conserved 5′-GGAG-3′ sequence and its CSD targeting an adjacent 5′-region. Binding of the third molecule of Lin28 to the high-affinity 5′-AUGAUAC-3′ site involves only its CSD and implicates relocating the CSD of the first molecule of Lin28. The order of assembly proposed for pre-let-7g may differ for precursors of other let-7 family members.

Based on available data, we propose a model for stepwise assembly of Lin28 on the terminal loop of pre-let-7g (Figure 7). In this model, the first molecule of Lin28 initiates complex formation through a high-affinity interaction between its NCp7-like domain and the 5′-GAGGG-3′ site at the 5′-bulge, which is followed by binding of its CSD to the nearby 5′-AUGAUAC-3′ site. Both the 5′-bulge and the NCp7-like domain have previously been identified as important determinants of the pre-let-7g/Lin28 interaction (18,19). Our stoichiometric binding assay further reveals that an intact 5′-bulge on pre-let-7g and the NCp7-like domain of Lin28 are essential for formation of a stable 1:1 complex and that disruption of both leads to random assembly of multimeric complexes. This supports the idea that they initiate the ordered assembly of the 1:1, 1:2 and 1:3 complexes, as previously suggested (19). The concept that the CSD contacts the 5′-AUGAUAC-3′ site (residues 16–21) in the 1:1 complex is supported by the high affinity of Lin28 towards the TL13-26 fragment containing this sequence as well as fluorescence quenching of 2-AP21 associated with CSD binding on formation of a 1:1 complex. Furthermore, the proposed model for the 1:1 complex matches remarkably well with RNase protection studies, where the 5′-GAGGG-3′ and the 5′-AUGAUAC-3′ sites are the most protected regions of pre-let-7g on binding of Lin28 (18). Interactions at these two RNA sites in the 1:1 complex occurs with the CSD and NCp7-like domain arranged in a 3′ to 5′ orientation that may not be preferred for Lin28 (51,57), but that may be dictated by the availability and high affinity of these sites on pre-let-7g. Binding at these sites destabilize Watson–Crick base pairs within the terminal loop, as previously observed (18), as both RNA domains are known to interact with single-stranded RNAs. Results from our 2-AP assay are also in agreement with RNA destabilization on formation of the 1:1 complex, whereas our FRET strand-displacement assay supports an RNA melting activity for Lin28, as previously suggested (52).

This terminal loop destabilization caused by the binding of the first Lin28 protein exposes a previously concealed high-affinity binding site on the 3′-strand, making it available for Lin28 binding (Figure 7). We propose that the NCp7-like domain of the second protein binds the conserved 5′-GGAG-3′ sequence, whereas its CSD binds an adjacent 5′-region, possibly the 5′-CGGUAC-3′ sequence, which shares a 5′-YGRUAC-3′ motif with the well-characterized 5′-AUGAUAC-3′ CSD binding sites of TL-let-7g (52). Binding of a second molecule of Lin28 at this proposed site is consistent with our 2-AP fluorescence assay, in which a Lin28-dependent change in 2-AP31 fluorescence correlates with formation of the 1:2 complex. We propose that the third molecule of Lin28 binds the high-affinity 5′-AUGAUAC-3′ site through its CSD. This would require the CSD of the first molecule of Lin28 to relocate, which is conceivable given its low sequence specificity and the flexibility of the linker region between the CSD and the NCp7-like domain (51,52). Furthermore, such repositioning of the first molecule of Lin28 is consistent with the increase in 2-AP5 fluorescence associated with formation of the 1:3 complex.

The proposed assembly mechanism may differ for precursors of other let-7 family members. For example, formation of the 1:1 complex with pre-let-7d likely involves the exposed 5′-GGAG-3′ binding site at the 3′-end of TL-let-7d, as previously reported (51,52), rather than the concealed 5′-AGGG-3′ binding site at the 5′-end of TL-let-7d. Moreover, multimeric assembly could also occur through alternative and likely less important pathways, as evident from the reduced ability of the C139A/C161A variant to form multimeric complexes with TL-let-7g.

The stepwise assembly proposed here underlines the ability of Lin28 to efficiently multimerize on pre-let-7 to form a 1:3 complex, previously noted with pre-let-7g (19) and pre-let-7a-1 (55). One could argue that the observed stoichiometry contradicts previous reports, where 1:1 binding was observed between mouse Lin28 and TL-let-7d by sedimentation equilibration ultracentrifugation experiments (51) and between human Lin28B and pre-let-7g using both size-exclusion chromatography and mass spectrometry (61). However, these previous studies were not performed under conditions where the protein is in excess of the RNA (51,61) and that allow binding of multiple copies of Lin28 to pre-let-7.

The stepwise assembly recapitulates other important aspects of Lin28 function. It involves the contribution of both RNA domains of Lin28, in agreement with their importance for binding target RNAs *in vitro* and for *in vivo* function (15,17,19,24,25,28,51,53,57,69,70). The NCp7-like domain initiates complex formation with an accessible high-affinity site to allow an orderly stepwise assembly of Lin28 on its target pre-miRNA. After the initial binding, the CSD melts the RNA to allow Lin28 to efficiently multimerize on the RNA. In the resulting 1:3 complex, all three Lin28-binding sites in the terminal loop are occupied by one molecule of Lin28, with the two G-rich sites near the Dicer cleavage sites forming high-affinity interactions. Notably, binding at these G-rich sites in the 1:3 complex involves both RNA domains of Lin28, with the CSD and the NCp7-like domain arranged in a 5′ to 3′ orientation on the RNA, in agreement with biochemical and X-ray structural studies (51) as well as transcriptome-wide studies of LIN28B-bound mRNA targets (57). Given that precursors of the let-7 family have large terminal loops in which these two important G-rich sequences are highly conserved (20,71), formation of multimeric complexes observed here with pre-let-7a-1 (55), pre-let-7d and pre-let-7g likely extends to other family members and may have important regulatory functions.

### Role of the stepwise assembly of Lin28 for in vivo regulation of pre-let-7 biogenesis

Lin28 is highly expressed in stem cells, progenitor cells as well as poorly differentiated tumours, and Lin28 levels decrease during differentiation (72). Assembly of multiple molecules of Lin28 on the terminal loop of pre-let-7 could therefore play important regulatory functions in cells where Lin28 is highly abundant. Here, we tested the possibility that multimerization of Lin28 on pre-let-7 RNAs, which is observed at high Lin28 concentrations, could modulate the activity of Dicer. We determined that binding of only one molecule of Lin28 on the terminal loop of pre-let-7g and pre-let-7d is sufficient to elicit maximum inhibition of Dicer cleavage, but that binding of multiple molecules of Lin28 on the terminal loop of pre-let-7a-1 is required to reach maximum inhibition of Dicer cleavage. Interestingly, recent studies in HEK293 cells indicate the let-7a (7a-1, 7a-2 and 7a-3) is not strongly regulated by Lin28 compared with other members of the let-7 family, including let-7d and let-7g (54,55). In these studies, Lin28 may have reached a level that strongly inhibited Dicer processing for several let-7 precursors, but that only weakly inhibited Dicer processing for let-7a precursors. In contrast, efficient Lin28-dependent inhibition of pre-let-7a-1 maturation was observed in other studies (16,17,27–29), including studies with mammalian embryonic stem cells, where higher levels of Lin28 may allow its multimerization on pre-let-7a-1 and more efficient Dicer inhibition.

*In vivo*, the specificity of the Lin28-dependent inhibition of let-7 biogenesis likely stems from several aspects of Lin28 function depending on the cell type, including its ability to specifically form a highly stable 1:1 complex with let-7 precursors, its ability to multimerize on these precursors and the coordination of its RNA-binding process with additional factors that control let-7 biogenesis. For example, the processive activity of the terminal uridylyl transferase TUT4 on pre-let-7 RNAs depends on the formation of a specific Lin28/pre-let-7 complex (17,28,31). By comparison, although Dicer likely competes with Lin28 for pre-let-7g binding, TUT4 must recognize a Lin28/pre-let-7 complex (17,28,31), and it is not yet clear if it can associate with the 1:1, 1:2 and/or 1:3 complex. Additional factors, including the Drosha-DGCR8 complex, hnRNPA1 (20,21), KSRP (21–23) and the Dis3L2 3′-5′ exonuclease (34,35) are present in vivo to assist Lin28 in modulating the production of mature let-7 as needed for development, cell differentiation and tumour suppression (5–13). Such factors may intervene at different steps of the assembly of Lin28 on pre-let-7 (or pri-let-7) terminal loops, either by competing with Lin28 for its binding sites or by associating with specific complexes along the assembly pathway. Multimerization of Lin28 on pre-let-7 terminal loops may inhibit the activity of factors that promote let-7 biogenesis and/or assist factors that prevent let-7 biogenesis, and thereby contribute to maintaining a pluripotent cell state. Similarly, the previously observed multimerization of Lin28 on target mRNAs may antagonize miRNA-mediated repression by displacing the miRNA-induced silencing complexes (36,69), although such multimerization may also contribute to recruit factors that control the fate of mRNAs. Thus, the present study provides a framework for future investigations aimed at better understanding the various functions of Lin28, particularly those that examine the interplay with additional factors involved in let-7 biogenesis.

## SUPPLEMENTARY DATA

Supplementary Data are available at NAR Online.

## FUNDING

Natural Sciences and Engineering Research Council of Canada (to P.L.); PhD scholarships from the Canadian Institutes of Health Research and the Université de Montréal (to A.D.); MSc scholarship from the Université de Montréal (to J.B.); a Canada Research Chair in Structural Biology and Engineering of RNA (to P.L.). Funding for open access charge: Natural Sciences and Engineering Research Council of Canada.

*Conflict of interest statement*. None declared.

## Supplementary Material

Supplementary Data
