# Supplementary material for: Stepwise assembly of multiple Lin28 proteins on the terminal loop of let-7 miRNA precursors
Source: Nucleic Acids Res. 2014 Jan 21;42(7):4615–28. doi: 10.1093/nar/gkt1391 (PMC3985620; doi:10.1093/nar/gkt1391)
Supplement: Supplementary Data [file supp_42_7_4615__index.html]

Stepwise assembly of multiple Lin28 proteins on the terminal loop of let-7 miRNA precursors — Stepwise assembly of multiple Lin28 proteins on the terminal loop of let-7 miRNA precursors — Supplementary Data 

# Stepwise assembly of multiple Lin28 proteins on the terminal loop of let-7 miRNA precursors

## Supplementary Data

files

**Files in this Data Supplement:**

- Supplementary Data - pdf file
