# Supplementary material for: Stepwise assembly of multiple Lin28 proteins on the terminal loop of let-7 miRNA precursors
Source: Nucleic Acids Res. 2014 Jan 21;42(7):4615–28. doi: 10.1093/nar/gkt1391 (PMC3985620; doi:10.1093/nar/gkt1391)
Supplement: Supplementary Data [file supp_gkt1391_nar-01755-y-2013-File009.pdf]

Stepwise assembly of multiple Lin28 proteins on the terminal loop of let-7 miRNA precursors

Alexandre Desjardins, Jonathan Bouvette and Pascale Legault

Département de Biochimie et Médecine Moléculaire, Université de Montréal, C.P. 6128, Succursale Centre-Ville, Montréal, QC, Canada, H3C 3J7

SUPPLEMENTARY MATERIAL

Supplementary Figure S1

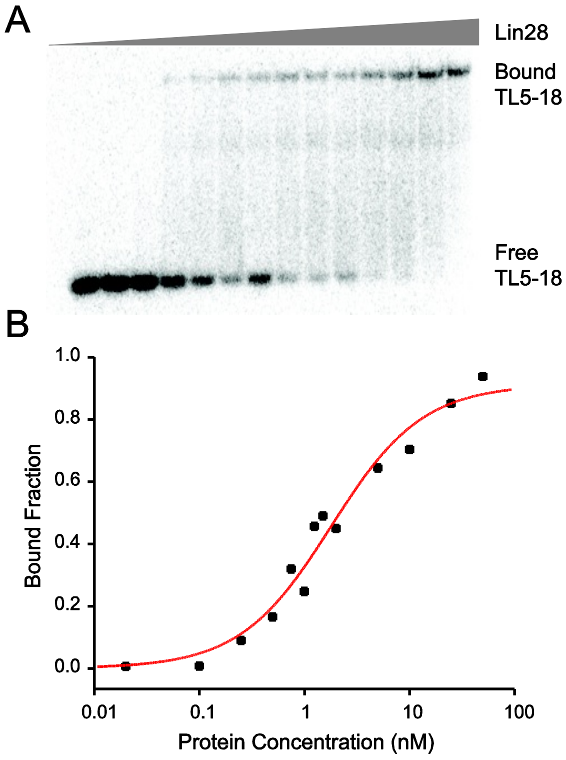

Supplementary Figure S1. Binding studies of TL-let-7g to Lin28 by EMSA for determination of the dissociation constant. (**A**) Typical EMSA performed with 1 pM 5'-[^{32}P]-labeled TL5-18 RNA and increasing concentrations of Lin28 (0.0, 0.02, 0.10, 0.25, 0.50, 0.75, 1.0, 1.25, 1.5, 1.75, 2.0, 5.0,10, 25 and 50 nM). (**B**) Fraction of bound RNA as a function of total protein concentration, fitted to the one site binding equation (red line; with K_d = 1.8 nM and R^2 = 0.97).

Supplementary Figure S2

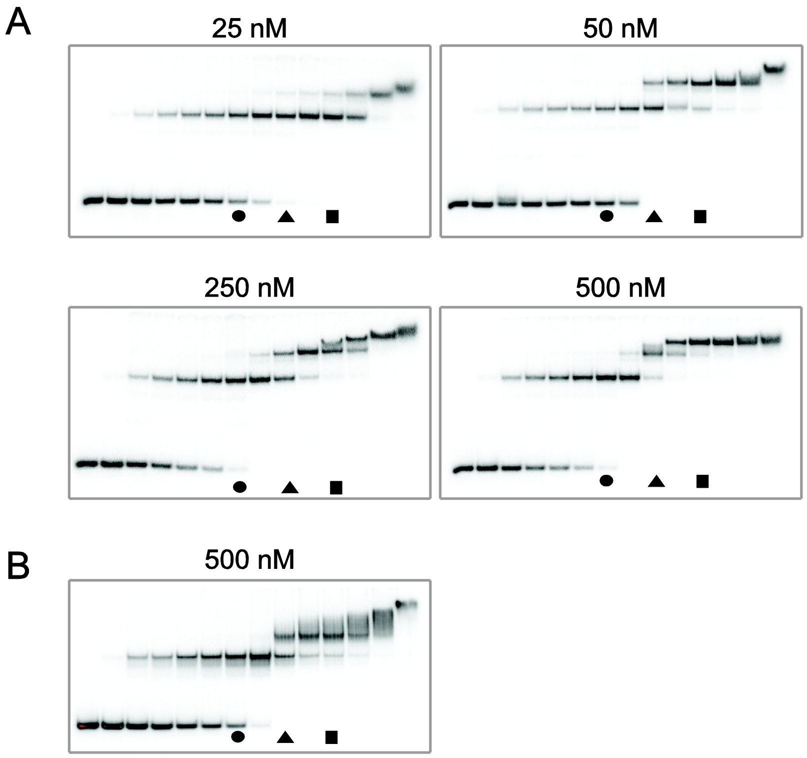

Supplementary Figure S2. Stoichiometric binding assay by native gel electrophoresis for Lin28 binding to different concentrations of TL-let-7g (**A**) and to 500 nM of pre-let-7g [for the exact sequence see (1)] (**B**). Each assay is performed with the indicated RNA concentration, including 10 pM 5'-[^{32}P]-labeled RNA, and increasing concentrations of protein (from 0.1X to 10X the RNA concentration). The gel lanes with RNA:protein ratios of 1:1.5, 1:2.5 and 1:3.5 are identified by a circle, a triangle and a square, respectively.

Supplementary Figure S3

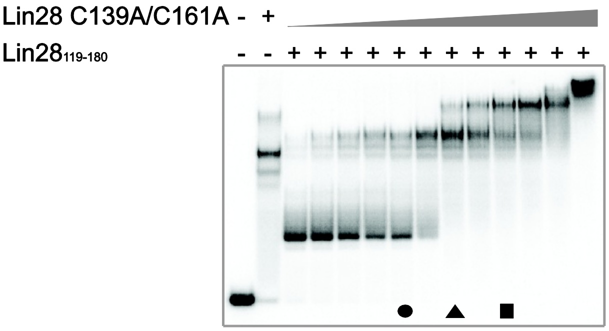

Supplementary Figure S3. Competitive stoichiometric binding assay by native gel electrophoresis between Lin28 C139A/C161A and Lin28$_{119\text{-}180}$ for binding to TL-let-7g. The assay is performed with 0.5 μM RNA, including 10 pM 5'-[^{32}P]-labeled RNA, increasing concentrations of Lin28 C139A/C161A (0.00, 0.05, 0.25, 0.375, 0.50, 0.625, 0.75, 1.00, 1.25, 1.50, 1.75, 2.00, 2.50 and 5.00 μM) and constant concentration of Lin28$_{119\text{-}180}$ (1 μM). A control assay was also performed with 1 μM Lin28 C139A/C161A (+) in the absence of Lin28$_{119\text{-}180}$ (-). The gel lanes with RNA:Lin28 C139A/C161A ratios of 1:1.5, 1:2.5 and 1:3.5 are identified by a circle, a triangle and a square, respectively.

Supplementary Figure S4

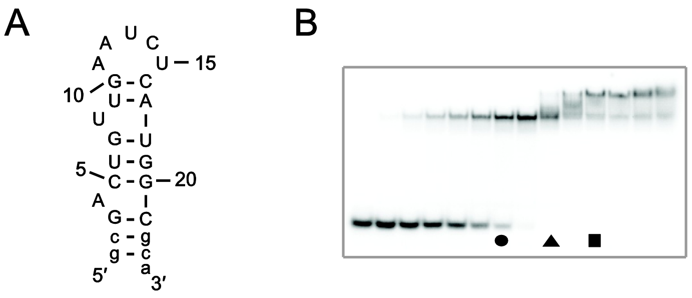

Supplementary Figure S4. Stoichiometric binding assay by native gel electrophoresis for Lin28 binding to TL-miR-21a. (**A**) Sequence and proposed secondary structure of TL-miR-21a. (**B**) Stoichiometric binding assay of TL-miR-21a with Lin28. The assay is performed with 0.5 μM RNA, including 10 pM 5'-[^{32}P]-labeled RNA, and increasing concentrations of protein (0.00, 0.05, 0.25, 0.375, 0.50, 0.625, 0.75, 1.00, 1.25, 1.50, 1.75, 2.00, 2.50 and 5.00 μM). The gel lanes with RNA:protein ratios of 1:1.5, 1:2.5 and 1:3.5 are identified by a circle, a triangle and a square, respectively.

Supplementary Figure S5

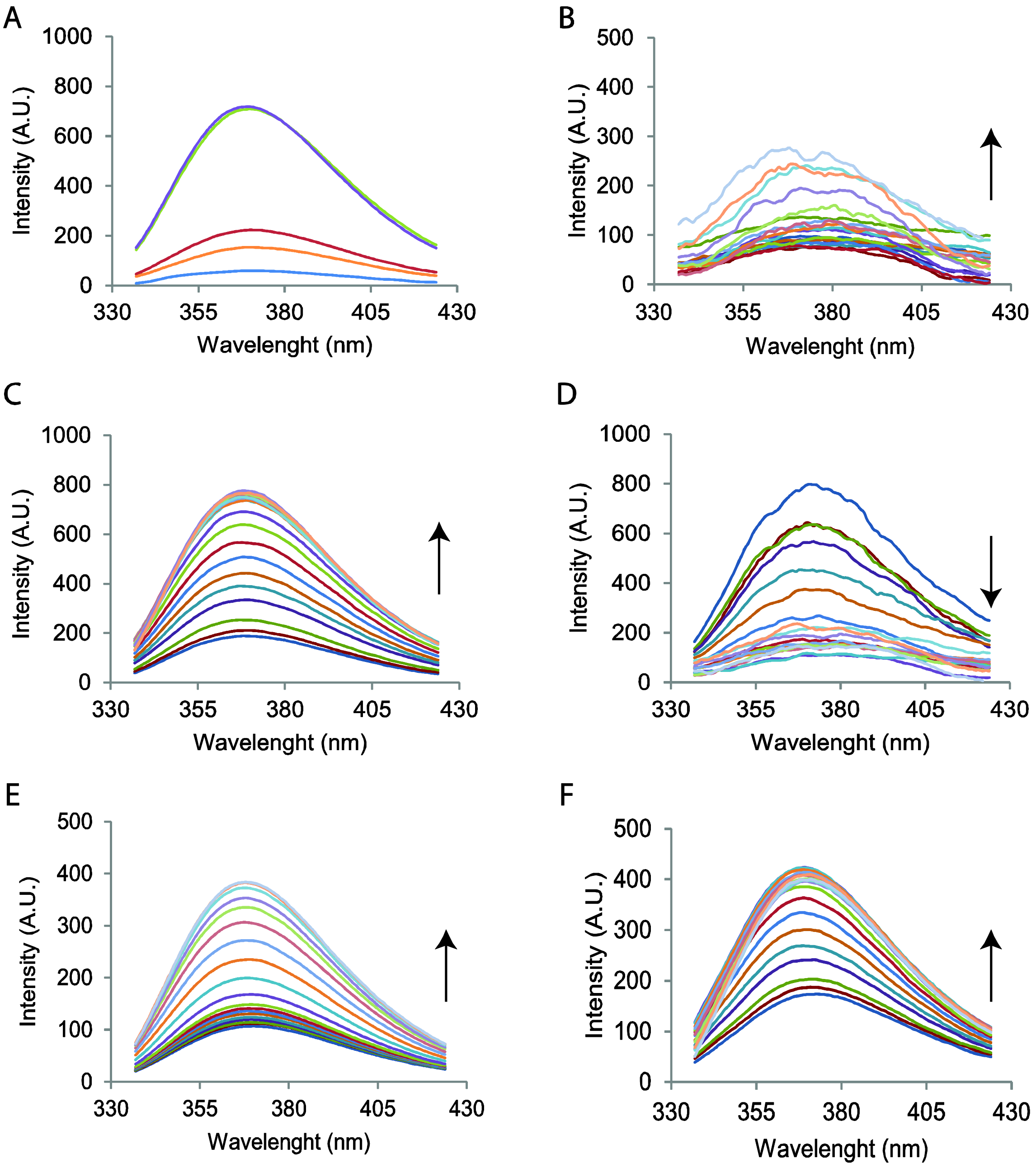

Supplementary Figure S5. Effect of addition of Lin28 on the emission spectra of 2-AP-modified TL-let-7g RNAs. (**A**) Emission spectra recorded in the absence of protein for TL-let-7g RNAs (500 nM) containing a single 2-AP at position A5 (blue), A9 (red), A21 (green), A31 (purple) or A36 (orange). Spectra were all recorded with the same detector gain settings for

comparison purposes. (**B**)-(**F**) Difference emission spectra following the addition of Lin28 (0 μM, dark blue; 0.050 μM, dark red; 0.125 μM, dark green; 0.250 μM, dark purple; 0.375 μM, dark cyan; 0.500 μM, dark orange; 0.625 μM, blue; 0.750 μM, red; 0.875 μM, green; 1 μM, purple; 1.125 μM, cyan; 1.250 μM, orange; 1.375, light blue; 1.500 μM, light red; 1.625 μM, light green; 1.750 μM, light purple; 1.875 μM, light cyan; 2 μM, light orange; and 2.5 μM, grey) to different TL-let-7g RNAs containing a single 2-AP at positions (**B**) A5, (**C**) A9, (**D**) A21, (**E**) A31 or (**F**) A36. The arrows indicate the increase (arrow up) or decrease (arrow down) of fluorescence emission upon addition of protein. The detector gain setting was optimized for each titration experiment, thus the intensities detected for different RNA-protein combinations cannot be directly compared.

Supplementary Figure S6

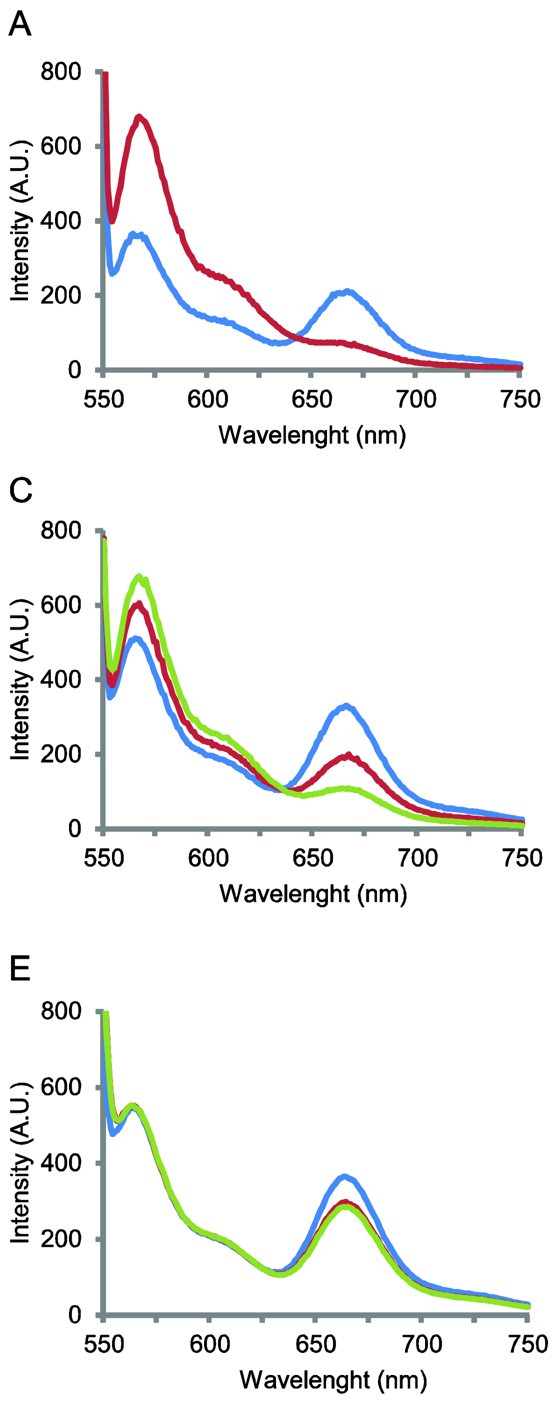

Supplementary Figure S6. Effect of addition of Lin28 on the emission spectra of Cy3/Cy5-labeled RNA duplexes. The RNA duplex containing a G-rich bulge ($duplex_{bulge}$) was titrated with (**A**) Lin28, (**C**) Lin28 C139A/C161A and (**E**) $Lin28_{119-180}$. The control complementary RNA duplex ($duplex_{comp}$) was also titrated with (**B**) Lin28, (**D**) Lin28 C139A/C161A and (**F**) $Lin28_{119-180}$. The emission spectra were recorded from 550/10 nm to 750/10 nm after excitation at 535/20 nm on samples containing 25 nM Cy3/Cy5-labeled RNA duplexes and either 0 nM (blue), 500 nM (red) or 2 μM protein (green). In (**A**) the 2-μM titration point was not recorded, whereas all three titration points are present in the other panels.

Supplementary Figure S7

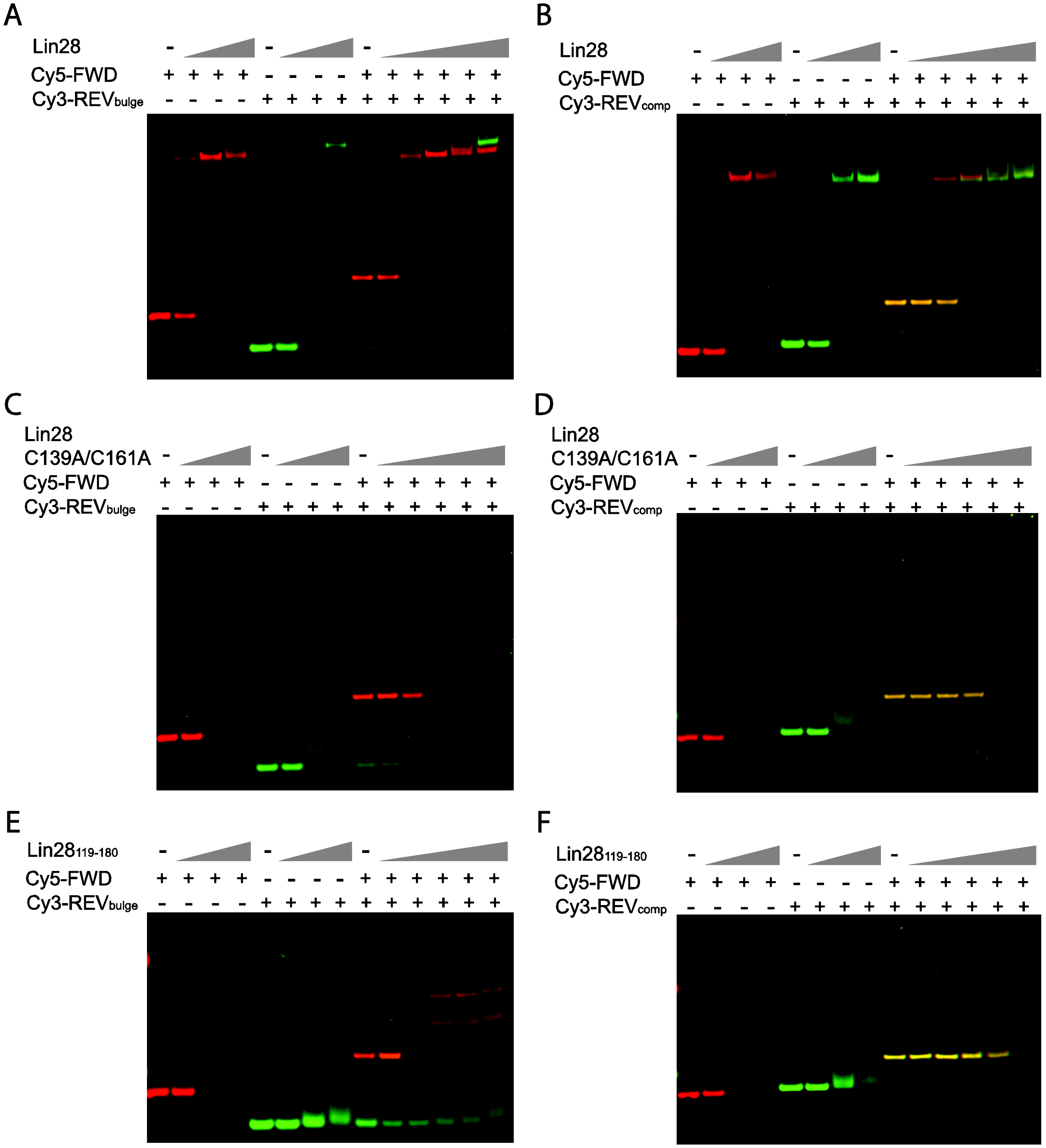

Supplementary Figure S7. Strand displacement assay monitored by native gel electrophoresis with 25 nM of Cy3/Cy5-labeled duplex RNAs. (**A**) Additions of Lin28 (0, 25, 250 and 2000 nM) to isolated RNA strands, either 25 nM of Cy5-FWD or 25 nM Cy3-REV_{bulge} followed by the additions of Lin28 (0, 25, 100, 250, 500 and 2000 nM) to 25 nM $duplex_{bulge}$ formed by annealing Cy5-FWD to Cy3-REV_{bulge}. Titrations similar to those described in (**A**) were performed with (**C**) Lin28 C139A/C161A and (**E**) $Lin28_{119\text{-}180}$. (**B**) Titrations were performed as in (**A**), except that Cy3-REV_{bulge} is replaced by Cy3-REV_{comp} and $duplex_{bulge}$ is replaced by $duplex_{comp}$. Titrations similar to those described in (**B**) were performed with (**D**) Lin28 C139A/C161A and (**F**) $Lin28_{119\text{-}180}$. The Cy3 and Cy5 signals are represented by green and red colors, respectively, and the superposition of both signals produces a spectrum of colors from yellow to orange-red. In (**B**), binding of Lin28 to the duplex can not be distinguished from binding to the individual strands. In several cases, binding of Lin28 creates such a diffuse band on the gel that the RNA seems to disappear from the gel.

Supplementary Figure S8

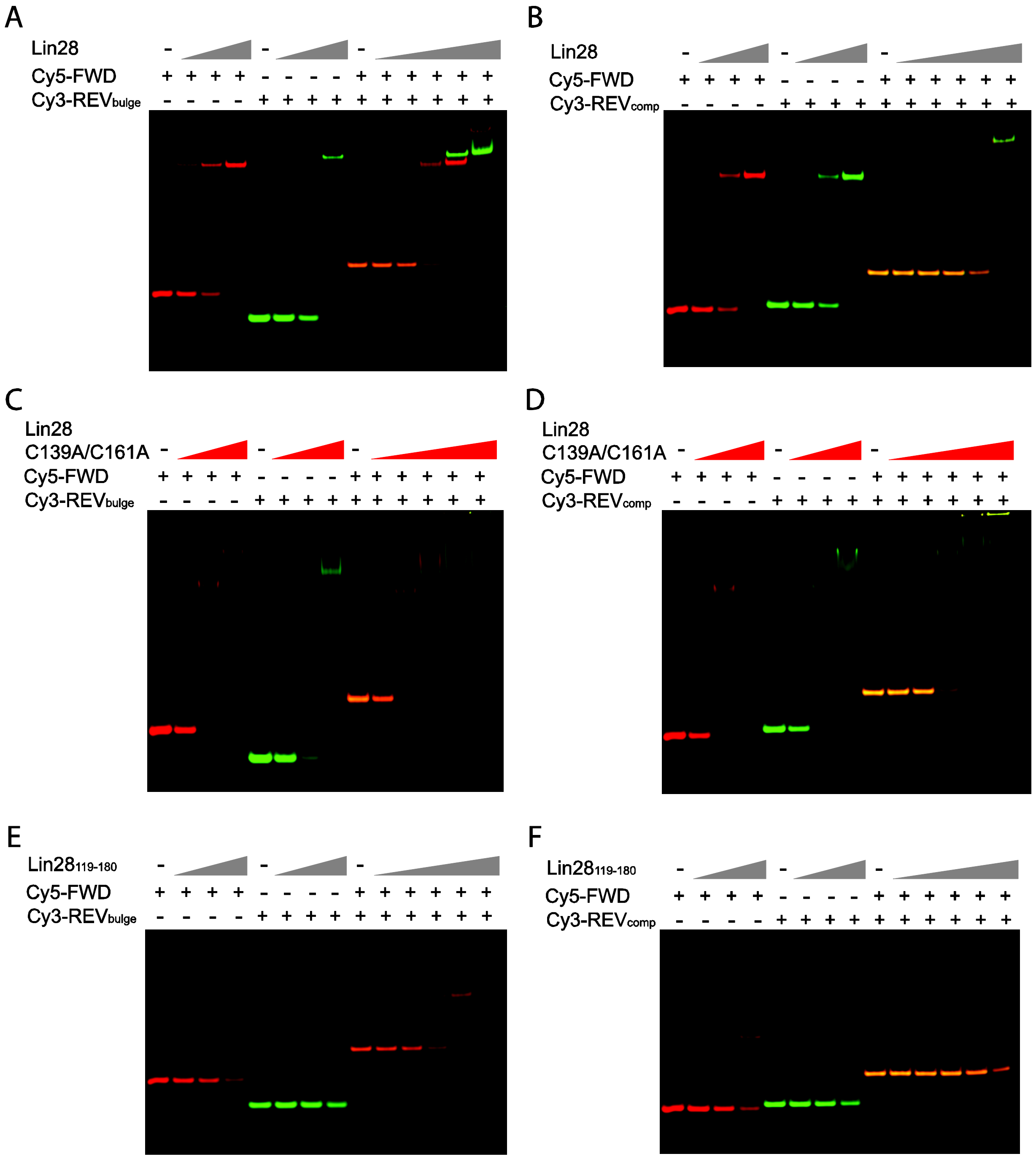

Supplementary Figure S8. Strand displacement assay monitored by native gel electrophoresis with 250 nM of Cy3/Cy5-labeled RNA. (**A**) Additions of Lin28 (0, 125, 250 and 500 nM) to isolated RNA strands, either 250 nM of Cy5-FWD or 250 nM Cy3-REV$_{bulge}$ followed by the additions of Lin28 (0, 125, 250, 500, 1000 and 2000 nM) to 250 nM Cy3/Cy5-labeled duplex$_{bulge}$ formed by annealing Cy5-FWD to Cy3-REV$_{bulge}$. Titrations similar to those described in (**A**) were performed with (**C**) Lin28 C139A/C161A and (**E**) Lin28$_{119\text{-}180}$, except that different protein concentrations (red gradients) were used in (**C**). **B**) Titrations were performed as in (**A**), except that Cy3-REV$_{bulge}$ is replaced by Cy3-REV$_{comp}$ and duplex$_{bulge}$ is replaced by duplex$_{comp}$. Titrations similar to those described in (**B**) were performed with (**D**) Lin28 C139A/C161A and (**F**) Lin28$_{119\text{-}180}$, except that different protein concentrations (red gradients) were used in (**D**). In (**C**) and (**D**) 0, 0.5, 1 and 2 μM of Lin28 were sequentially added to the isolated RNA strands and 0, 0.5, 1, 2, 5 and 10 μM of protein were sequentially added to the duplex RNA (red gradients). The Cy3 and Cy5 signals are represented by green and red colors, respectively, and the superposition of both signals produces a spectrum of colors from yellow to orange-red. In (**B**), binding of Lin28 to the duplex can not be distinguished from binding to the individual strands. In several cases, binding of Lin28 creates such a diffuse band on the gel that the RNA seems to disappear from the gel.

Supplementary Figure 9

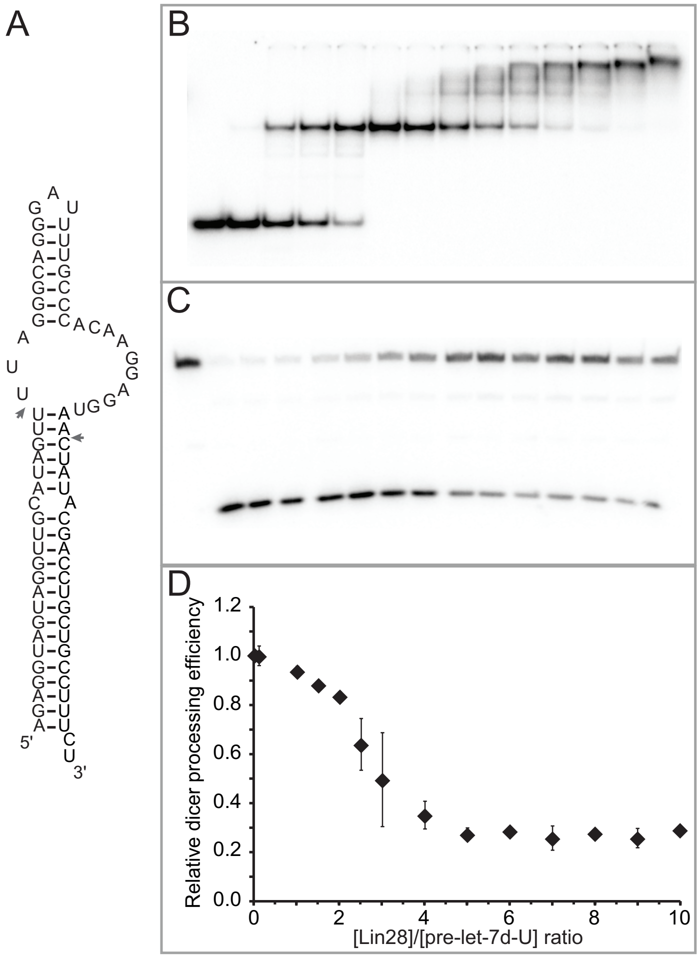

Supplementary Figure S9. Dicer processing assay of pre-let-7d-U. (**A**) Primary and proposed secondary structures of the pre-let-7d-U RNA. Dicer cleavage sites are identified with arrows. (**B**) Stoichiometric binding assay detected by native gel electrophoresis for Lin28 binding to pre-let-7d-U. Each assay is performed with 0.5 μM 5'-phosphorylated RNA, including 40 pM 5'-[^{32}P]-labeled RNA and increasing concentration of protein (0.00, 0.05, 0.50, 0.75, 1.25, 1.50, 2.00, 2.50, 3.00, 3.50, 4.00, 4.50 and 5.00 μM). (**C**) Dicer processing assay detected by denaturing gel electrophoresis of pre-let-7d-U under varying Lin28 concentrations. The first well contains 0.5 μM RNA without Lin28 and Dicer. The subsequent wells are for the Dicer assay performed under the same conditions as for the stoichiometric binding assay (in **B**), but with an additional 0.25 U of Dicer enzyme. (**D**) Relative Dicer processing efficiency plotted against Lin28/pre-let-7d-U concentration ratios (n=3).

Supplementary Figure 10

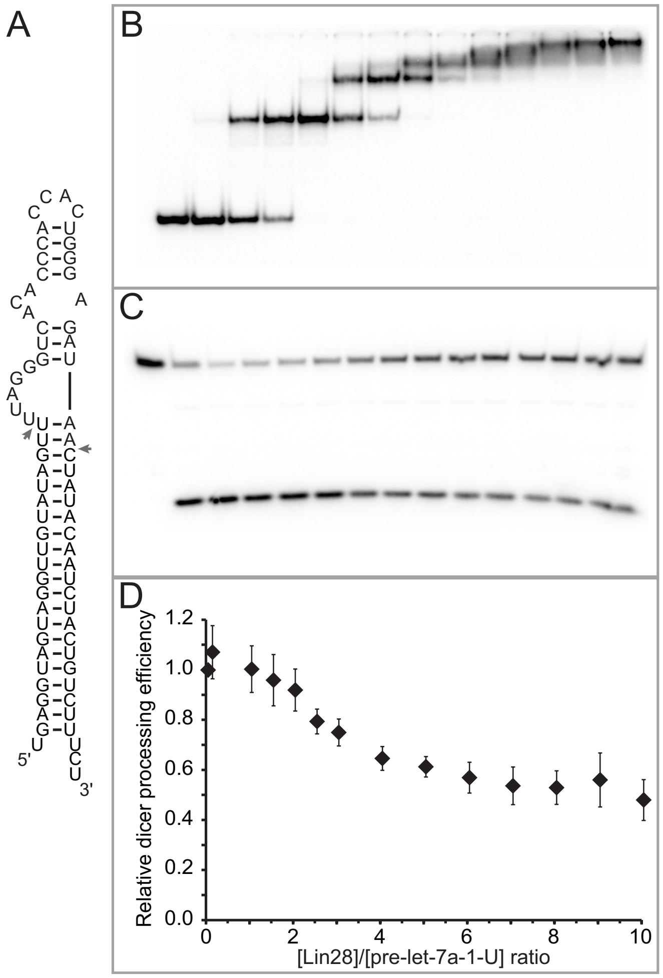

Supplementary Figure S10. Dicer processing assay of pre-let-7a-1-U. (**A**) Primary and proposed secondary structures of the pre-let-7a-1-U RNA. Dicer cleavage sites are identified with arrows. (**B**) Stoichiometric binding assay detected by native gel electrophoresis for Lin28 binding to pre-let-7a-1-U. Each assay is performed with 0.5 μM 5'-phosphorylated RNA, including 40 pM 5'-[^{32}P]-labeled RNA and increasing concentration of protein (0.00, 0.05, 0.50, 0.75, 1.25, 1.50, 2.00, 2.50, 3.00, 3.50, 4.00, 4.50 and 5.00 μM). (**C**) Dicer processing assay detected by denaturing gel electrophoresis of pre-let-7a-1-U under varying Lin28 concentrations. The first well contains 0.5 μM RNA without Lin28 and Dicer. The subsequent wells are for the Dicer assay performed under the same conditions as for the stoichiometric binding assay (in **B**), but with an additional 0.25 U of Dicer enzyme. (**D**) Relative Dicer processing efficiency plotted against Lin28/pre-let-7a-1-U concentration ratios (n=4).

REFERENCE

1. Desjardins, A., Yang, A., Bouvette, J., Omichinski, J.G. and Legault, P. (2012) Importance of the NCp7-like domain in the recognition of pre-let-7g by the pluripotency factor Lin28. *Nucleic Acids Res*, **40**, 1767-1777.
